# A New Empirical Model for Viscosity of Sulfonated Polyacrylamide Polymers

**DOI:** 10.3390/polym11061046

**Published:** 2019-06-14

**Authors:** Saeed Akbari, Syed Mohammad Mahmood, Hosein Ghaedi, Sameer Al-Hajri

**Affiliations:** 1 Petroleum Engineering Department, Universiti Teknologi PETRONAS, Seri Iskandar, Tronoh 32610, Malaysia; ensamyo87@gmail.com; 2 Shale Gas Research Group (SGRG), Institute of Hydrocarbon Recovery, Petroleum Engineering Department, Universiti Teknologi PETRONAS, Seri Iskandar, Tronoh 32610, Malaysia; 3 School of Environment, Tsinghua University, Beijing 100084, China; ghaedi.hosein63@gmail.com

**Keywords:** rheology, sulfonated polyacrylamide, design of experiment (DOE), response surface methodology (RSM), empirical model

## Abstract

Copolymers of acrylamide with the sodium salt of 2-acrylamido-2-methylpropane sulfonic acid—known as sulfonated polyacrylamide polymers—had been shown to produce very promising results in the enhancement of oil recovery, particularly in polymer flooding. The aim of this work is to develop an empirical model through the use of a design of experiments (DOE) approach for bulk viscosity of these copolymers as a function of polymer characteristics (i.e., sulfonation degree and molecular weight), oil reservoir conditions (i.e., temperature, formation brine salinity and hardness) and field operational variables (i.e., polymer concentration, shear rate and aging time). The data required for the non-linear regression analysis were generated from 120 planned experimental runs, which had used the Box-Behnken construct from the typical Response Surface Methodology (RSM) design. The data were collected during rheological experiments and the model that was constructed had been proven to be acceptable with the Adjusted R-Squared value of 0.9624. Apart from showing the polymer concentration as being the most important factor in the determination of polymer solution viscosity, the evaluation of the model terms as well as the Sobol sensitivity analysis had also shown a considerable interaction between the process parameters. As such, the proposed viscosity model can be suitably applied to the optimization of the polymer solution properties for the polymer flooding process and the prediction of the rheological data required for polymer flood simulators.

## 1. Introduction to Polymer Flooding

Although a continuous depletion of oil reservoir pressure during primary recovery can eventually result in a fall in oil production, this can be overcome by injecting the water into the reservoir via an immiscible displacement process called water flooding. The Buckley-Leverett method [1] can be defined as the simplest and most broadly employed approach to determine the advancement with regards to the fluid displacement front for an immiscible displacement process.

In improved oil recovery techniques, the mobility ratio (*M*) is known to play an important role, where its calculation can be shown as such:

(1) $$M=\frac{Mobility\;of\;diplacinf\;fluid\;\left(water\;in\;here\right)}{Mobility\;of\;diplaced\;fluid\;\left(oil\;in\;here\right)}=\frac{k_{w}/\mu_{w}}{k_{o}/\mu_{o}}$$

where k, μ denote effective permeability and viscosity, respectively. The sub-scripts o and w refer to oil and water, respectively. Expression of fractional flow to water ($f_{w}$) based on Equation (1) is then attained where $q_{w}$ and $q_{o}$ denote volumetric flow rate of water and oil phases, respectively:

(2) $$f_{w}=\frac{q_{w}}{q_{w}+q_{o}}=\frac{1}{1+\frac{1}{M}}$$

**Figure 1:**
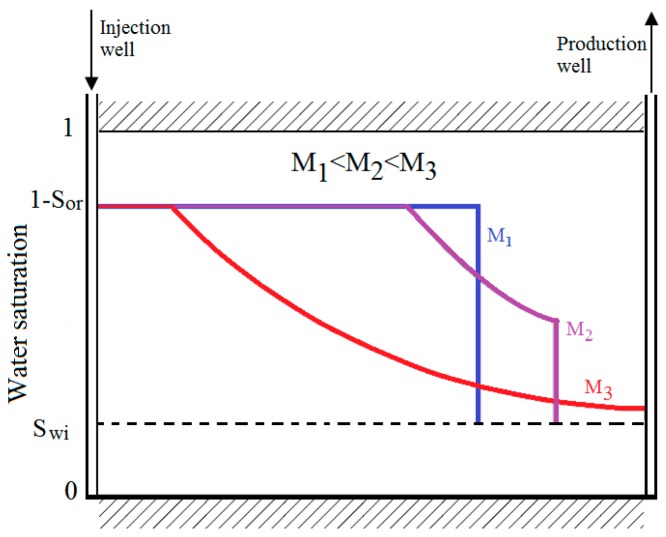
Water saturation profile of different mobility ratio (*M*).

The above expression indicates that the water fractional flow $\left(f_{w}\right)$ at any point in the reservoir had been highly affected by the mobility ratio, where a lower mobility ratio is shown to result in a lower $f_{w}$ value and vice-versa. The three saturation profiles in a water flooding process of three different mobility ratios are shown in Figure 1. As shown by the figure, a reduction in *M* can lead to a more piston-like displacement with higher Buckley-Leverett front heights and consequently, higher recovery efficiency.

**Figure 2:**
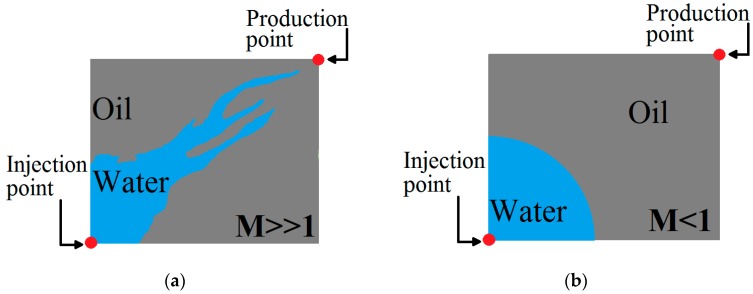
Illustrative cases for (**a**) unstable and (**b**) stable displacement.

Apart from the fractional flow theory, with an increase in *M*, there is a chance to introduce instability in the fluid displacement process. This phenomenon results in lower viscosity water’s fingering into the oil, which is considered a very inefficient areal sweep. This is also referred to as viscous fingering [2]. Figure 2a shows the viscous fingering issue pertaining to mobility ratios that are greater than 1. Once a channel is reached by a finger and subsequently the production well, sweeping the remaining oil becomes problematic. This is due to the injected water opting for the flow path that was set by the finger. Figure 2b demonstrates a case where mobility ratio is lesser than 1. Here, the water front grows in a manner that is stable and radial where no finger gets ahead of the front. Finally, this gives a better sweep regarding the reservoir.

The polymer injection method was first introduced in the early 1960s as a way of mitigating the low-saturation water front and unstable displacement issues during water flooding. Addition of polymer to the injected water in a process known as polymer flooding can greatly increase the viscosity of injected water [3,4,5,6]. This viscosity increment results in lowering the mobility of water phase relative to the oil (see Equation (1)) and hence stabilize the areal flood and, at the same time, improve the microscopic displacement efficiency [7,8,9]. Polymer flooding has been utilized commercially in the petroleum industry for over four decades [10].

Synthetic polymers (e.g., partially hydrolysed polyacrylamide, known as HPAM) have been used successfully for viscosity improvement in actual polymer flooding projects [11,12]. HPAM was found to have faced degradation issues because of the amide groups severely hydrolysis to carboxylates under temperature levels exceeding 70 °C. Apart from that, the high salinity reservoirs with more than 40,000 ppm of total dissolved solids (TDS) had also resulted in HPAM precipitation and consequently, a decline in the viscosity level [13,14,15].

The chemical instability of the HPAM at temperatures higher than 70 °C can be overcome by co-polymerizing the acrylamide (AM) with the monomer groups, such as that of 2-Acrylamido-2-Methylpropane Sulphonate (AMPS), which are hydrolysis-resistant [13,16,17,18]. Regarding the rheological aspect, the previous study [18] had shown the introduction of a sulfonic acid unit in the molecular chain have led to the increased chain rigidity in sulfonated polyacrylamide copolymer (AM/AMPS copolymer) and hence, creating a better shear-resistance as compared to that of the HPAM. As such, the AM/AMPS copolymer had demonstrated its potential of being used in the polymer flooding process, where they can be utilized for reservoirs with a temperature level as high as 90 °C without being subjected to the risk of thermal degradation [13,16,17,18,19,20].

Apart from the essential thermal stability criterion, it is also essential to develop an empirical model for the bulk solution viscosity for a successful application of the sulfonated polyacrylamide polymers for enhanced oil recovery (EOR) purposes. The proposed model has to consider the effects of polymer properties (i.e., molecular weight, sulfonation degree), reservoir conditions (i.e., reservoir temperature, salinity and hardness concentrations) as well as other relevant operational variables (i.e., polymer concentration, shear rate, temperature and time) on the polymer solution viscosity. The specific objective of this work is to develop such a model for the bulk viscosity of sulfonated polyacrylamide copolymers. The developed model then can be incorporated into a polymer flooding simulator for predicting accurate polymer solution viscosity in different conditions. Prior to the launch of a field trial, the economic viability of a polymer flooding project needs to be analysed, which is based on the simulation results.

## 2. Suitable Viscosity Model for Polymer Flooding Simulation

Polymer solutions exhibit both Newtonian and non-Newtonian properties at different flow stages. They typically exhibit Newtonian behaviour at very low and very high shear rates. Additionally, a region of shear thinning where the viscosity of the fluid decreases exists at moderate share rates. The viscosity share rate relationship, $\mu \left(\dot{\gamma }\right)$, has been empirically modelled in one or more of these regions [21,22,23,24,25,26,27]. The power law model, which describes the pseudoplastic region, is undoubtedly the most commonly applied analytical form of $\mu \left(\dot{\gamma }\right)$ and is expressed as follows [28]:

(3) $$\mu \left(\dot{\gamma }\right)=K{\left(\dot{\gamma }\right)}^{n-1}$$

where *K* and *n* are constants. In the case of a Newtonian fluid, *n* = 1, while *K* is just a viscosity constant. Although Equation (3) had adequately described the pseudo-plastic region, it could not be suitably used at high and low shear rates. Subsequently, Carreau’s equation has been reported as a more general model for these shear regimes [9,22,23]. The Carreau equation expresses the viscosity function as follows:

(4) $$\mu \left(\dot{\gamma }\right)=\mu_{\infty }+\left(\mu_{0}-\mu_{\infty }\right){\left[1+\left(1+{\left(\lambda \dot{\gamma }\right)}^{2}\right)\right]}^{\left(n-1\right)/2}$$

where $\mu_{\infty }$, $\mu_{0}$, λ and *n* denote infinite shear rate viscosity, zero shear rate viscosity, a time constant (the inverse of a shear rate at which nonlinearities begin to dominate) and a fitting parameter, respectively. However, despite providing an improved fit of viscometric data over a wide range of shear rates, Carreau’s equation had the required information on four parameters as opposed to the two as stated in the Power law. 

The economic potential of polymer flood is mostly determined by simulation and has been studied by many researchers [29,30,31]. Commercial reservoir simulators such as CMG-STARS (Computer Modelling Group), ECLIPSE and the University of Texas Chemical Flooding Simulator (UTCHEM) are some of the most commonly available models used in the evaluation of this complex EOR process.

The above models were found to have serious limitations in their use as viscosity models for polymer flooding simulation purposes. It is because they had not considered the effects of polymer concentration, salinity/harness concentration, temperature and aging time on the viscosity level. A winning viscosity model for polymer flooding application should, therefore, consider these parameters because of several reasons. Firstly, the polymer concentration is known to vary during polymer transportation in porous media as a result of adsorption and diffusion, which can largely affect the polymer solution viscosity. Secondly, since the injection water may have a different composition from those of the formation water, their mixture in the reservoir may generate a brine with a variable ionic strength as a function of time. Finally, the aging time effect has to be included in the viscosity model since the injected polymer phase may take months to reach production well. It is noteworthy that there may be a possibility of a more pronounced aging time effect on the polymer viscosity at high-temperature levels.

The polymer flood model of the UTCHEM simulator is deemed to be more robust than the other simulators as it had considered the salinity and the hardness effects on polymer viscosity as well as the polymer concentration and shear rate effects [30]. The viscosity of the polymer solution at a low (near zero) shear rate, $\mu_{p}^{0}$, in UTCHEM model is calculated as a function of polymer and electrolyte concentrations by using the modified Flory–Huggins model [32] as shown in Equation (5).

(5) $$\mu_{p}^{0}=\mu_{w}\left(1+(A_{p1}C_{p}+A_{p2}C_{p}^{2}+A_{p3}C_{p}^{3}\right)C_{SEP}^{sp}$$

where $A_{p1}$, $A_{p2}$ and $A_{p3}$ are the empirical constants that have been deduced from the experimental data of a given polymer. $\mu_{w}$ is the viscosity of solvent the before adding polymer and $C_{SEP}$ is the effective salinity that is used in the polymer property calculations and is composed of the following definition:

(6) $$C_{SEP}=\frac{C_{5}+\beta_{P}C_{6}}{C_{1}}$$

where $C_{5}$, $C_{6}$ and $C_{1}$ are the respective anion, divalent and water concentrations in the aqueous phase, while $\beta_{P}$, a value that is obtained in the laboratory, is used to determine the effects of divalent cations on the effective salinity for the polymer. The effect of both monovalent and divalent cations are equal when $\beta_{P}$ is equal to one. However, a stronger effect of the divalent cations on the polymer properties will be shown if the $\beta_{P}$ value is greater than one. As for the term sp, this denotes the input parameter for determining polymer viscosity as a function of salinity and is assumed to be the slope of a straight line under the log-log plot of $\frac{\mu_{p}^{0}-\mu_{w}}{\mu_{w}}$ versus $C_{SEP}$ [30,33,34].

The polymer viscosity at a low shear rate, $\mu_{p}^{0}$, as calculated from Equation (5) will become the input parameter for estimating the viscosity at higher shear rates. The effect of the shear rate on polymer viscosity is modelled by Meter’s model [35] and is shown as Equation (7):

(7) $$\mu_{p}=\mu_{w}+\frac{\mu_{p}^{0}-\mu_{w}}{1+{\left(\frac{\dot{\gamma }}{{\dot{\gamma }}_{1/2}}\right)}^{p_{\alpha }-1}}$$

where ${\dot{\gamma }}_{1/2}$ is the shear rate at which the viscosity is the average of $\mu_{p}^{0}$ and $\mu_{w}$. $p_{\alpha }$ is an empirical coefficient.

Polymer viscosity is highly dependent on the shear rate. If the flow behaviour in a porous medium is described by using the bulk viscosity measured at various shear rates, then it is necessary to calculate the equivalent shear rate to that in the bulk viscometer. This is denoted as the in-situ shear rate (${\dot{\gamma }}_{eq}$), which is modelled according to the modified Blake Kozeny capillary bundle equation, which can be as expressed as Equation (8):

(8) $${\dot{\gamma }}_{eq}=\frac{{\dot{\gamma }}_{c}\left|{\vec{u}}_{w}\right|}{\sqrt{\bar{k}k_{rw}\emptyset S_{w}}}$$

where ${\dot{\gamma }}_{c}$ ($s^{-1})$ is the empirical shear rate coefficient that is derived from the laboratory experiments, while $\bar{k}$, $k_{rw}$, ∅, $S_{w}$ and ${\vec{u}}_{w}$ are the corresponding average permeability, relative permeability of the water, porosity, water saturation and water phase velocity.

Although the viscosity model evaluation in the UTCHEM simulator had shown consideration of more parameters than Carreau’s equation, it had, however, suffered from the consequences of using the one factor at a time (OFAT) approach. By using this approach, a mismatch of model parameters could be observed between the high and low polymer concentrations, where the solutions with a lower polymer concentration were found to have exhibited Newtonian behaviour in most of the shear rate ranges and not the shear thinning behaviour as shown at the higher polymer concentration level [36].

Apart from the UTCHEM viscosity model, several other empirical correlations for viscosity determination were also developed based on the OFAT approach [4,5,37]. However, OFAT is found to be ill-suited for modelling a process with interactions [38]. There is interaction when the impact of one variable on the response relies on the level of another variable. As such, the design of experiment (DOE) can be considered as an alternative approach to OFAT in the model development of such processes, where it had considered and managed the interactions between variables. The UTCHEM model as discussed above is not only technically cumbersome, it had also disregarded the interaction that had taken place between the variables and in some circumstances, may lead to its model failure when large impacts are created as a result of these interactions. Such interactions are common amongst the variables in polymer flooding process [38].

Soft computation technique such as Artificial Neural Network (ANN) and neuro-fuzzy, as a combination of artificial neural networks and fuzzy logic, also have been successfully used to model polymer solution viscosity [39,40]. The main problem for this type of modelling is its black box nature, which makes it difficult (if not impossible) to incorporate them into the polymer flooding simulator. Therefore, it is desired to have a white model or an equation. This problem may be overcome by presenting the black box model in the form of matrices which has not been done so far and can be a subject of future research.

The literature review showed that the process variables such as polymer concentration, shear-rate, sulfonation degree, mono/divalent ions concentration and molecular weight have considerable influence on the polymer solution viscosity [9,17,20,34,41]. One of the main objectives of this work is to precisely assess and model the effects of the above-mentioned parameters on the polymer solution viscosity. It had been shown that not only scads of experiments are required to accommodate for the increasing number of process parameters under the classical design of the experiment, the utilization of a full factorial design would also be a highly time-consuming and arduous method to use [42]. Since it would be impractical to develop a large-scale DOE plan that is based on the 3^k^ full factorial design for *k* quantitative variables and the involvement of a large number of experiments (6561 runs), more efficient designs had thus been adopted in the study of the parameters. 

For this reason, the Response Surface Methodology (RSM), which is a specified technique of the DOE, had been designed to determine the optimal response for a specified range of factors. This design can also fit into a second order prediction equation to be employed for the response [43]. The Box-Behnken design (BBD), which is a specific type of RSM design, is a fraction of the 3^k^ designs equipped with additional centre points for design balance [44]. The BBD, which can be applied for systems with 3 to 10 number of factors, had been proven to be efficient even with the fewer numbers of experimental runs than to those of a 3^k^ design [42,45]. Under this design type, three levels should be provided for each model inputs (parameters), namely minimum, middle, maximum, coded as −1, 0 and +1. Being rotatable (or “nearly” rotatable) is an important feature of this design [46]. In this study, the BBD was used to develop a viscosity model as a function of the process parameters.

## 3. Materials and Methods

### 3.1. Model Development Procedure Using DOE

The eight independent factors that had been identified under the literature review [9,17,20,34,47] as having possible significant effects on the polymer solution viscosity (polymer concentration, salinity and hardness concentrations, temperature, aging time and mole percent of the sulfonated repeating units known as sulfonation degree, molecular weight and shear rate) were chosen as input parameters for the model. The investigation ranges as well as the justifications of the selected range of each model input are depicted in Table 1.

There had been several reasons for considering the above-mention factors as model inputs. Firstly, factors such as salinity and hardness concentrations, molecular weight, as well as shear rate are directly related to the hydrodynamic size of the polymer (or alignment of the polymer molecules in the case of shear rate) and thereby affecting the solution viscosity. Secondly, the degree of polymer hydrolysis was also discovered to have been highly influenced by the sulfonation degree and aging time at high-temperature levels. The hydrolysis of the acrylamide moieties to the negatively charged acrylate can initially cause an increase in viscosity because of intra-chain charge repulsion. Finally, the alteration of polymer concentration changes the probability of interaction and entanglement between polymer chains and thereby highly affecting the polymer solution viscosity.

In this study, Box-Behnken design (BBD) was used instead of the full-factorial method. Based on the eight numerical factors and the considering no blocks, an experimental matrix of 120 runs was then generated by applying the BBD as the DOE strategy (given in Table S1 in Supplementary File A). In this context, run denotes the act of operating the process with the required factors at a certain stipulated level. 

Each experimental run was designed based on the conditions imposed by the experimental matrix. The polymer solution samples that had been prepared and aged according to the required time and the predetermined temperature was brought in for viscosity measurement. These measured viscosities and the respective run settings were then imported and tabulated as the response data shown in Table S1 of Supplementary File A.

#### 3.1.1. Confounding Evaluation

When two variables are confounded (or coupled) by changing one variable, the other changes with it so that the true cause of any change in the output cannot be determined [48]. In this study, the presence of confounding or correlated variables was investigated through the computation of a Pearson’s coefficient. Equation (9) shows the Pearson’s coefficient (r) between factors X and Y for all observations (i’s) [49]:

(9) $$r=\frac{\sum_{i=1}^{n}X_{i}Y_{i}-\frac{1}{n}\sum_{i=1}^{n}X_{i}\sum_{i=1}^{n}Y_{i}}{\sqrt{\sum_{i=1}^{n}X_{i}{}^{2}-\frac{1}{n}{\left(\sum_{i=1}^{n}X_{i}\right)}^{2}}\sqrt{\sum_{i=1}^{n}Y_{i}{}^{2}-\frac{1}{n}{\left(\sum_{i=1}^{n}Y_{i}\right)}^{2}}}$$

Numerically, the value of the Pearson’s coefficient is in the range of −1 to +1. A perfect positive relationship between two variables gives a value of +1. Conversely, a perfect negative correlation is signified by a value of −1.

#### 3.1.2. Input Factor Coding and Response Transformation

Coded variables have been employed for all of the model fittings. This can be attributed to the use of engineering units that yields different numerical results and often, the results’ interpretation is more tedious and difficult versus the coded unit analysis. Furthermore, with regards to the coded variable analysis, the impact of altering each design factor over a one-unit interval could be easily compared and achieved by examining the obtained model coefficients’ magnitude [42]. In this study, Equation (10) had been employed to code each of the factor $x_{i}$ to the three levels (−1, 0, 1) as follows:

(10) $$x_{i}=\left(X_{i}-X_{0}\right)/\Delta X$$

where $X_{0}$ is the value of $X_{i}$ (selected factor) at the centre point, while ΔX represents the step change.

From the results of the experimental runs, the viscosity was observed to have a range of 1.18 to 59.46 cp (mPa.s) with a maximum to minimum ratio shown as 50.38. Since a ratio with a greater than 10 value usually indicates the need for a transformation, Equation (11) was thus applied to transform response data.

(11) $$y^{\prime }=ln\left(\frac{y-lower}{upper-y}\right)=ln\left(\frac{y}{1000-y}\right)$$

where y and $y^{\prime }$ are the respective response values before and after the transformation. In Equation (11), the values for *lower* and *upper* are stand for the expected lowest and highest y values. In this study, the zero value was selected for the *lower* value, while 1000 had been selected as the *upper* value since it would be unlikely for the viscosity of the sulfonated polyacrylamide solution to be zero and the polymer solution viscosity to be as high as 1000 cp (mPa.s) in the selected ranges for the model inputs (factors). 

#### 3.1.3. Model Fitting and Validation

After the output values (measured responses) for each run had been inserted in the designed table, a non-linear regression analysis was then used to find a fitted empirical model to the data set through the use of the Design Expert software (version 11.00) [50]. 

In the model development stage, conducting a continuous assessment on the root mean square error (R^2^) and normality tests on the residuals, the differences between the prediction model and the actual value (observed value), were necessities to be assured of staying away from under and over-fitting issues from occurring [42,48].

#### 3.1.4. Sensitivity and Uncertainty Analysis

Sensitivity analysis is used to assess the sensitivity level of the model output (polymer solution viscosity) to the changes in the model inputs. The results obtained from the sensitivity analysis can also be used to assess the contribution of the input parameters to the uncertain analysis outcomes.

In this study, the Sobol method is used to perform the sensitivity analysis and to determine the relative parameter effect on the model response. The ability to provide a global sensitivity analysis, effective handling of non-linear responses, as well as the measurement of the interaction effects and quantification of the measures in percentages are some of the advantages of the Sobol method. The Sobol sensitivity analysis was conducted using the SimLab software (version 2.2).

Uncertainty analysis, on the other hand, is performed when there is a need to see how the model output for a specific run (or condition) is influenced by the uncertainty of model inputs. The input probability distribution for each input parameter can be derived from experience and can take a uniform, triangle, Gaussian (normal), log-normal forms. For this study, the normal error distribution for each input parameter was selected based on the experimental observations and analysis was performed using NIST Uncertainty Machine and considering Monte Carlo simulation.

### 3.2. Experimental Details

#### 3.2.1. Characteristics of the Sulfonated Polyacrylamide Polymers 

**Figure 3:**
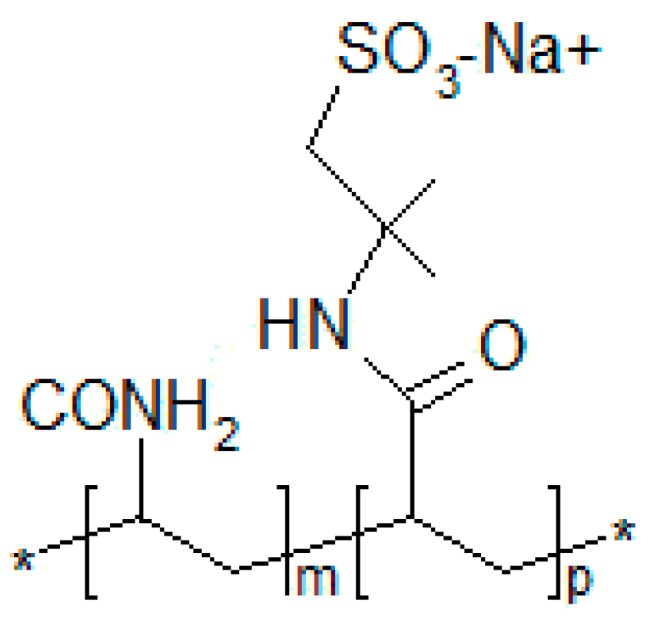
Structure of sulfonated polyacrylamide.

All of the sulfonated polyacrylamide polymers that were examined in this study had been provided in powder form from an industrial production workshop of SNF Floerger (ANDREZIEUX Cedex, France). The general structure of these copolymers, as well as their respective polymers and compositions, are shown in Figure 3 and Table 2, respectively. 

#### 3.2.2. Polymer Solution Preparation

For samples that were required to be evaluated for determining the impact of salinity and hardness concentrations, the addition of NaCl (sodium chloride) and CaCl_2_ (calcium chloride) was done to the deionized water in suitable proportions to produce solutions in the desired concentrations (based on experimental design). Sodium chloride (NaCl, 99.5%) and Calcium Chloride (CaCl_2_, >98%) were purchased from Merck KGaA (Darmstadt, Germany). The PF3XXXXM1 Purelab Flex 3 water purification System made by ELGA (High Wycombe, United Kingdom) was used to polish pre-purified water to Ultrapure water. The prepared brine was placed under a propeller stirrer and rotation at 700 rpm started just before adding polymer powder. The sample beakers were covered with aluminium foils to reduce solution contact with air. Thirty minutes after addition of the last polymer, setting the stirrer speed to medium (300 rpm) was done and stirring of the solution is done overnight to guarantee completion of hydration as well as homogenization of the polymer solution. The prepared samples of 10 mL were then placed into 30-mL vials and the lid was closed post addition of 3 wt % IBA (isobutyl alcohol) as an antioxidant. To make the vials air-tight, applying of silicone glue was done.

#### 3.2.3. Viscosity Measurement

The prepared polymer solutions were then transferred to an oven with a pre-set temperature level (determined by experimental design) and stored at different times as per the aging requirement of the test. The viscosity measurement was then conducted by adhering to the following steps. The sample vial was first taken out from the oven and rapidly cooled to 25 °C to avoid the possible occurrence of water evaporation and unstable viscosity readings at higher temperature levels. The rheological profiles were then obtained at aerobic condition using the Bohlin Gemini 2 (Malvern Instruments, Malvern, UK) with a cone-plate geometry (1°, 4 cm) at a shear rate interval of 1 s^−1^ to 100 s^−1^, where the temperature of a rheometer geometry system was controlled by the Temperature Controlling Unit.

#### 3.2.4. Oxygen-Free Environment

Most reservoirs possess a reducing environment and the dissolved oxygen was not found in the produced water. In this research work, the addition of iso-butyl-alcohol (IBA) was done to establish an oxygen-free condition, which helped in avoiding polymer oxidation. Due to easy oxidization of the alcohol, it acts as a sacrificial agent to safeguard the polymer against oxidization [6,17,52,53]. IBA ((CH₃)₂CHCH₂OH, >99%) was purchased from Merck KGaA (Darmstadt, Germany).

## 4. Results and Discussion

This section presents the results from each of the steps that was taken during the development of a viscosity model for sulfonated polyacrylamide (AM/AMPS) polymers. Once the model had been developed and validated, the results were then derived from trend analysis and the interaction plots.

### 4.1. Confounding Evaluation

The evaluation of the confounding effect between factors marks the first step after data collection. As shown by the correlation matrix of factors in Table 3, the Pearson’s coefficient (*r*) between every two factors had demonstrated no signs of correlation between the factors (no confounding) as indicated by their zero coefficients (or close to zero for molecular weight and sulfonation degree).

### 4.2. Model Selection 

The statistical hypothesis tests such as those of *p*-value (probability value), lack of fit test as well as the R-squared (*R^2^*) values had shown a linear model even with the inclusion of the factor interaction terms (2 FI), was incapable of explaining the variation of the responses. In other words, the model was revealed to be under-fitted (due to the low value of adjusted R-squared; 0.87 for the linear model and 0.90 for 2 FI model). Whereas, the results showed that the cubic model was aliased.

Therefore, at first, a quadratic model covering all interaction terms was considered. To algorithmically choose the terms to be kept in the model, the automatic model selection was employed. *p*-value was the criterion and the backward selection was applied for the model-terms reduction method. *p*-value is regarded as the standard method to look for significant terms for retention and eliminate insignificant terms from the model (*p*-value > 0.1, refer to [48] for more details). 

The resulting ANOVA (presented in Table 4) for the reduced quadratic model had outlined the analysis of variance of the viscosity as a response. In this table, the adequacy measures of *R*^2^, adjusted *R^2^* and predicted *R^2^*, as well as an adequate precision ratio of 54.912, indicate that an acceptable model of discrimination had been achieved. The analysis had also shown the elimination of the interactions’ terms and the *Sd*^2^, *M_w_*^2^, *T*^2^, *At*^2^ as the quadratic terms had not influenced the attainment of the significant model. Here, the Sd, Mw, Hc, Sc, Pc, T, At and ShR had denoted the respective sulfonation degree, molecular weight, hardness concentration, salinity concentration, polymer concentration, temperature, aging time and shear rate. 

The prediction R-Squared of 0.9525 had been in reasonable agreement with the adjusted R-Squared of 0.9624 with a difference of less than 0.2. Adequate precision measures the signal to noise ratio and will be deemed as desirable if the ratio exhibits a value that is greater than 4. In this study, the resulted ratio of 54.91 had thus indicated the existence of an adequate signal. As such, the final empirical model in terms of the coded factors is shown as Equation (12).

(12) $$Ln\left(\frac{\mu }{1000-\mu }\right)=-5.21+\theta_{1}+\theta_{2}+\theta_{3}$$

(13) $$\begin{array}{l} \theta_{1}=-0.16\left(Sd\right)+0.40\left(Mw\right)-0.17\left(Hc\right)-0.052\left(Sc\right)+1.20\left(Pc\right)+0.070\;\left(T\right)\\ \;+\;0.046\left(At\right)-0.21\left(ShR\right) \end{array}$$

(14) $$\begin{array}{l} \theta_{2}=-0.094(Sd*Pc)-0.095\left(Mw*Sc\right)+0.20\left(Mw*Pc\right)-0.19\;\left(Mw*ShR\right)\\ \;+\;0.27\left(Hc*Sc\right)+0.088\left(Hc*ShR\right)-0.071\left(Sc*Pc\right)-0.19(Pc\\ \;*\;ShR) \end{array}$$

(15) $$\theta_{3}=0.11{\left(Hc\right)}^{2}+0.17{\left(Sc\right)}^{2}-0.30{\left(Pc\right)}^{2}+0.14{\left(ShR\right)}^{2}$$

The predictive model that is developed based on the coded factors can be employed to measure the relative impact of the factors via comparison of the factor coefficients. In this regard, the significant factors can be arranged in the following descending order: *Pc > Mw > Pc*^2^ *>* (Hc∗Sc) *>ShR >* (Mw∗Pc) *>* (Pc∗ShR) *>* (Mw∗ShR) *> Sc*^2^ *> Hc > Sd > ShR*^2^ *> Hc*^2^ *>* Mw∗Sc *>* Sd∗Pc *>* Hc∗ShR *>* (Sc∗Pc) > *T* > *Sc* > *At*. 

Equation (16) shows the final model in terms of the actual factors, which can be used to make a response prediction for the given levels of each factor at their original units.

(16) $$Ln\left(\frac{\mu }{1000-\mu }\right)=-6.814+\beta_{1}+\beta_{2}+\beta_{3}$$

(17) $$\begin{array}{l} \beta_{1}=-4.32\times 10^{-3}Sd+0.096Mw-5.07\times 10^{-4}Hc-0.095Sc+1.35\times 10^{-3}Pc\\ \;+\;4.67\times 10^{-3}T+3.06\times 10^{-3}At-2.37\times 10^{-3}ShR \end{array}$$

(18) $$\begin{array}{l} \beta_{2}=-4.82\times 10^{-6}\;\left(Sd*Pc\right)-3.82\times 10^{-3}\left(Mw*Sc\right)+2.71\times 10^{-5}\left(Mw*Pc\right)\\ \;-\;7.50\times 10^{-4}\;\left(Mw*ShR\right)+3.70\times 10^{-5}\;\left(Hc*Sc\right)+1.18\\ \;\times \;10^{-6}\;\left(Hc*ShR\right)-9.87\times 10^{-6}\left(Sc*Pc\right)-2.65\\ \;\times \;10^{-6}\left(Pc*ShR\right) \end{array}$$

(19) $$\beta_{3}=+4.94\times 10^{-8}Hc^{2}+7.11\times 10^{-3}Sc^{2}-1.42\times 10^{-7}\;Pc^{2}+5.69\times 10^{-5}ShR^{2}$$

where the units for Sd, Mw, Hc, Sc, Pc, T, At and ShR are mole percent, MDa, ppm, wt.%, ppm, °C, days and 1/s, respectively. 

It is noteworthy that the viscosity model has to be precise if an accurate estimation of the polymer flooding performance is desired. From the author’s observation in the polymer flooding simulation studies, changing the polymer solution viscosity for 3 cp (mPa.s) had resulted in a considerable effect on the oil recovery factor (although it might be case sensitive). As such, although Equation (12) is highly recommended in the determination of bulk polymer solution viscosity, certain end-users still prefer using simpler yet more error-prone models. Hence, starting with the least significant factor, each of the terms was then removed from Equation (12) to serve this purpose. The resulting models in terms of the coded factor, as well as their associated errors, are provided in Table S1 of Supplementary File B.

### 4.3. Model Validation

Apart from the error indices that were discussed earlier, the diagnostic plots can also provide useful tools for ensuring the model’s accuracy and the fulfilment of the ANOVA assumptions. Figure S1 of Supplementary File C shows four of these diagnostic plots which confirms that all of the assumptions required in the validation of the regression model seemed to have been fulfilled. 

### 4.4. Model Sensitivity and Uncertainty Analysis

**Figure 4:**
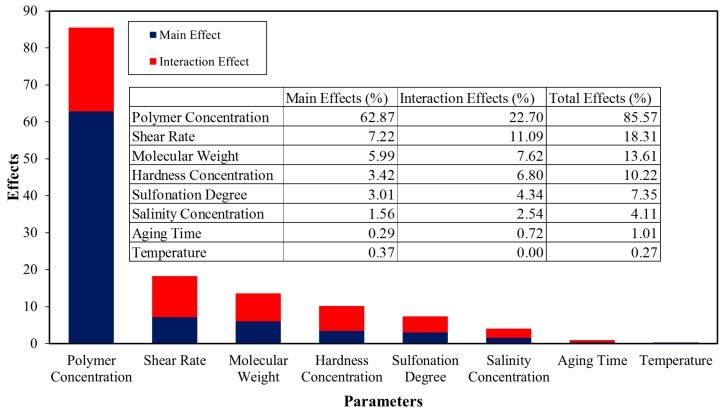
Sobol sensitivity analysis of eight input parameters of the viscosity model.

Figure 4 shows the results of the Sobol sensitivity analysis on the polymer viscosity model with the 8 model inputs. The Sobol sensitivity analysis model states that, the higher the percentage, the more sensitive the response will be towards that parameter. As seen from the results, the polymer concentration was revealed to be the most important contributing parameter, where it had influenced ~86% of the model output variability and was followed by the shear rate, molecular weight, hardness concentration, sulfonation degree, salinity concentration, aging time and temperature levels. A high percentage of polymer concentration in the sensitivity analysis is expected since the increase in polymer concentration will easily lead to higher interaction between the polymer macromolecules and consequently, sharper viscosity increment. 

The figure also shows that the sensitivity of the polymer solution viscosity had been more inclined towards the divalent ions than monovalent ions. An accepted explanation of this is that there is a strong bond between the carboxylate group (–COO^−^) and the divalent ions. A study conducted by Rashidi et al. [18] had also agreed with this observation, where the viscosity for both the HPAM and all of the studied sulfonated polyacrylamide polymers (AM/AMPS copolymer) were found to have decreased as a result of changing the solvent of 5 wt % NaCl to synthetic seawater (SSW) with divalent ions (although both solvents had the same ionic strength).

The experimental errors that had occurred during the measurement of model inputs may result in some uncertainty levels on the model output. For this reason, the uncertainty analysis can be used to quantify the associated risk of the reported model output’s value.

**Figure 5:**
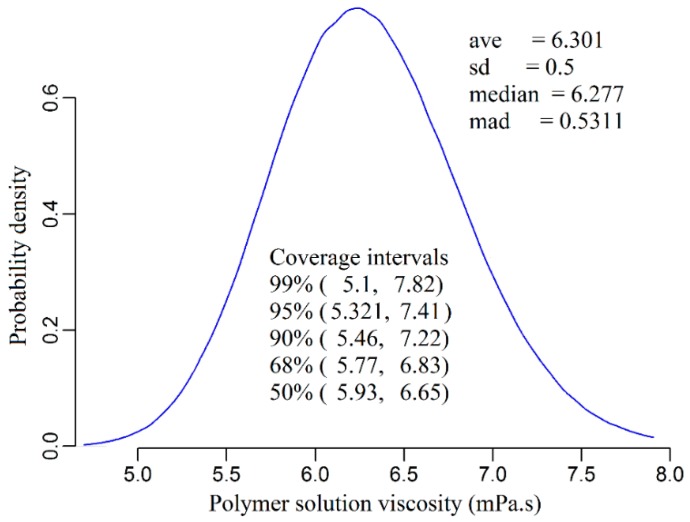
Probability density of the viscosity model output quantity.

The values of the model inputs for run number 28 in Table S1 (in Supplementary File A), as well as their associated measured uncertainties, are shown in Table 5. It is important to note that this run had been randomly selected for the uncertainty analysis purpose. By taking the given uncertainties and the normal error distribution of the model inputs into consideration, the probability distribution density of the polymer solution viscosity is then plotted as shown in Figure 5. The average viscosity value of the polymer solution was estimated to be 6.3 cp with a 0.5 cp standard deviation, while the uncertainty of the viscosity value was calculated to be 2 cp with a 95% confidence of interval.

### 4.5. The Main Effect of Factors on the Viscosity (One-Factor Plot)

Once the model had been established, a trend analysis was then used to interpret the model’s performance and explain the process behaviour. 

**Figure 6:**
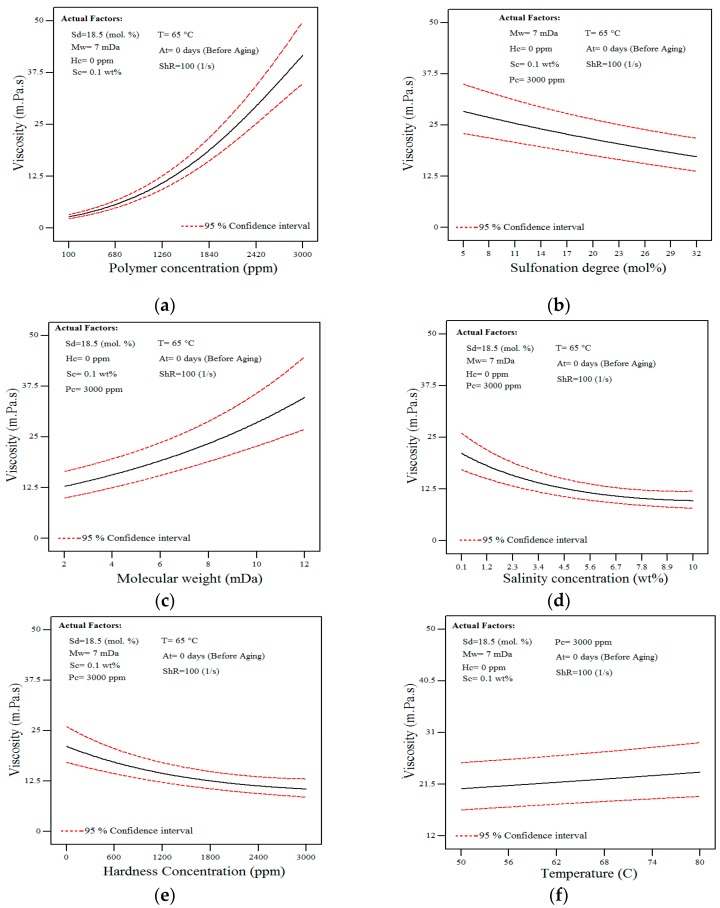

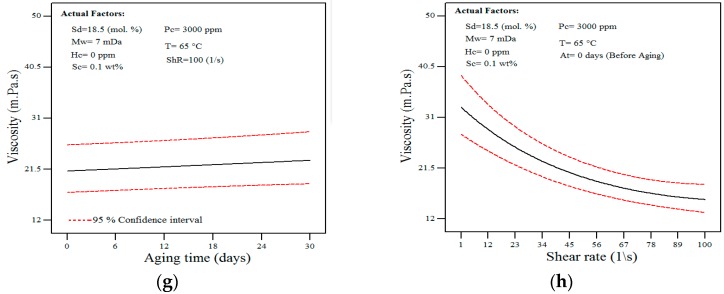
Model prediction for solution viscosity as a function of (**a**) polymer concentration, (**b**) sulfonation degree, (**c**) molecular weight, (**d**) salinity concentration, (**e**) hardness concentration, (**f**) temperature, (**g**) aging time and (**h**) shear rate.

In this regard, one of the primary plots is the main effect plot (one-factor plot) which is constructed by predicting the response over a range of a factor, from the low to high levels of a factor, while holding all other variables constant. The plotted graphs on all the considered factors are depicted in Figure 6 with their explanation presented in the subsequent paragraphs. Generally, the one-factor graph should be considered in the main effects analysis of factors that are not involved in an interaction. In this research, however, a discussion will be made on the main effect plots of all the factors regardless of their involvement in the interaction. 

**Figure 7:**
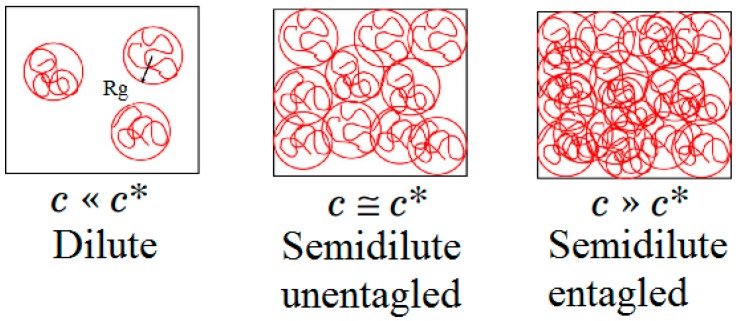
Different regimes based on polymer concentration (*C*) and overlap concentration (C*).

Figure 6a, shows that the increase in polymer concentration had resulted in higher viscosity levels. As shown in Figure 7, the increment of viscosity had been due to the fact that more chains are available at higher concentrations thus increasing the probability of interaction and entanglement between polymer chains [38]. Polymer molecules (in the form of spheres) are not in contact with each other at low concentrations. By increasing polymer concentration, polymer spheres become congested and ultimately touch each other at a so-called overlap concentration (C*) [54].

It can be concluded from the observed curvature and the numerical coefficient given in Equation (12) that the second order of polymer concentration (*Pc^2^*) had been a significant factor for determining the viscosity level. In fact, as a result of increasing the polymer concentration, the polymer regimes had changed from a diluted regime (C < C*) to semi-dilute and finally to a concentrated regime (C >> C*). Under the dilute regime, the C value had been below the C* (C: *C* << C*), therefore indicating the chains had behaved more or less independently with no to interact with each other. Furthermore, since the polymer chain had interacted primarily with the solvent molecules, the solution is regarded as being close to an ideal mixture. At *C* ≅ C*, the mobility of the polymer solution is greatly reduced in comparison with the dilute solutions since chains are overlapped and entangled. In a *semi-dilute regime that C* is above C* (C: *C* > C*), the situation is different and *the* viscosity is a strong function of the polymer concentration level. 

**Figure 8:**
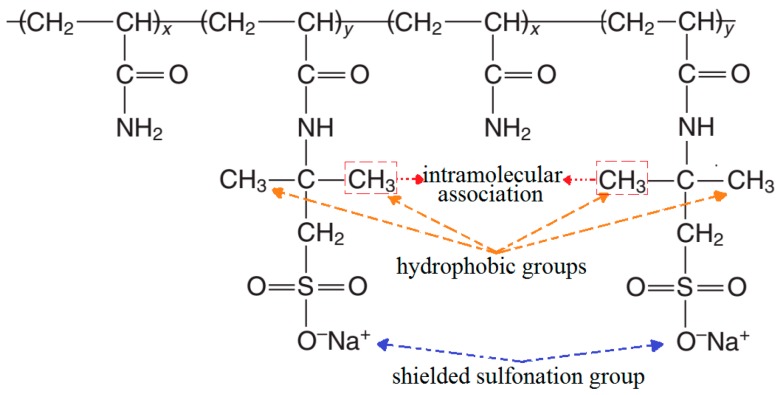
Hydrophobic groups association mechanism.

Figure 6b, shows that a decrease in the apparent solution viscosity is observed with increasing levels of the sulfonation degree. This may be because, in the presence of salt, the intra-molecular repulsion between negative charges of the sulfonated groups is minimized due to the shielding effect of counter ions (Na^+^) from the salt. As reported earlier [55], the reduction in the intra-molecular repulsion had therefore magnified the interaction between the hydrophobic groups (such as CH_3_) that were located along with the hydrophilic groups (AMPS) (Figure 8) in yielding a coil-like configuration. Since a higher level of sulfonation degree will result in a higher number of hydrophobic groups (CH_3_), there is, therefore, a greater chance of the intra-molecular association occurring, hence resulting in the reduction of the polymer hydrodynamic size and consequently, the reduced viscosity level.

Figure 6c shows that the increasing molecular weight had resulted in a higher viscosity level, which could be explained by the increasing chain interaction (also reported in previous studies [9,18,19,34]) facilitated by the higher chain sizes (radius of gyration, *Rg)*. 

The profitability of a polymer project can be achieved if the targeted viscosity level can be attained with low polymers concentration in the solution. Although polymers with high molecular weight are usually appropriate to be used for this purpose, they are however more susceptible and sensitive to shear degradation and mechanical entrapment as opposed to those with lower molecular weights. Ideally, the selected molecules should have minimal retention ability in a porous media for it to flow effortlessly through narrow channels without being blocked [47,56,57,58].

Figure 6d, shows that the increasing salinity concentration had resulted in a lower viscosity level, which had been reported earlier [18]. In distilled water, the molecules of the polymer have the potential to become expanded entirely because of the repulsive forces between the similarly charged groups along the polymer chain. By increasing salinity, most of the negative charges on the polymer chain will be neutralized by Na^+^ ions, which shrinks the polymer size and ultimately results in viscosity reduction. According to researchers [9,32,34,59,60], with an increase in salinity, there is a proportional increase in the overlap concentration (C*). This is possibly an outcome of the screening of charges on the polymer chain, which results in a reduction of the degree of expansion and causes the coiling of the polymers [18].

From the results shown in Figure 6d and Equation (12), the second order of salinity concentration (Sc^2^) had shown to be significant. It is because, above a critical salinity, all negative charges in the polymer structure will be shielded [18] and the addition of more salinity will not change the hydrodynamic size of the polymer in the solution and thus no further reduction will be observed in the viscosity level. 

Figure 6e, shows that the increasing hardness concentration had led to a lower viscosity level since the increase of the hardness level (Ca^2+^ ions) will neutralize most of the negative charges on the polymer chain and lead to the reduction of the viscosity level [9,34]. As shown in Figure 6e and Equation (12), the second order of hardness concentration (Hc^2^) was found to be a significant term with a critical concentration level. The same explanation that was given for the salinity effect in the previous paragraph can also be used to justify this observation. 

Figure 6f, shows that the increasing temperature had resulted in higher viscosity, although the effect had not been that highly significant. Since aging polymer at a higher temperature level may lead to a higher degree of hydrolysis and thereby higher intra-chain charge repulsion [60], polymer viscosity can increase by the time [11,20,61]. During the hydrolysis process as a result of thermal degradation, the amide functional group converts to a carboxylate group which is negatively charged. Introducing more negative groups facilitates more chain interaction and causes higher viscosity level (as discussed in the previous studies [18,19]). Apart from the hydrolysis of the amide groups, it is also worth mentioning that, for the case of sulfonated copolymers, the AMPS co-monomer groups may also hydrolyse at pH 8 and at temperature levels that are higher than 100 °C [62,63]. Polymer hydrolysis is caused by the intrinsic instability of molecules even in the absence of oxygen or other attacking species [34]. If the hydrolysis had only led to a chemical change of acrylamide moieties to the negatively charged acrylate and not the breakdown of the polymers, an increment of the viscosity level would then be observed.

Figure 6g, shows that the aging of polymer samples at 65 °C within 30 days had increased the solution viscosity linearly. The aging of polymer samples at a temperature level above 50 °C can facilitate the polymer hydrolysis that is a slow temperature-dependent process. With hydrolysis, more negatively-charged carboxylate groups will be introduced into the polymer structure and in turn, increases the intra-molecular repulsion. Consequently, the hydrodynamic size of the polymer will increase because of the higher repulsion and hence, resulting in the higher viscosity level (more details are provided in the literature [18,19]).

Figure 6h shows that increasing the shear rate from 1 to 100 (1/s) would result in a lower viscosity level. In this case, the reduction in viscosity could be observed from the aligned polymer molecules under the shear application (in accordance with [18,55,64]). This molecular alignment had allowed an easier flow of the molecules, hence reducing the viscosity level at higher shear rates since the increase of the shear rate and applied force will allow the polymer molecules to be oriented in the direction of the flow. However, a maximum orientation of polymer will occur after a specific shear rate (in upper-Newtonian regime), where no further viscosity reduction will be observed with the additional increase of the shear rates. This phenomenon had been the main reason for the observed curvature in Figure 6h and the existence of the second order of shear rate (ShR^2^) in Equation (12).

### 4.6. Interaction between Factors

**Figure 9:**
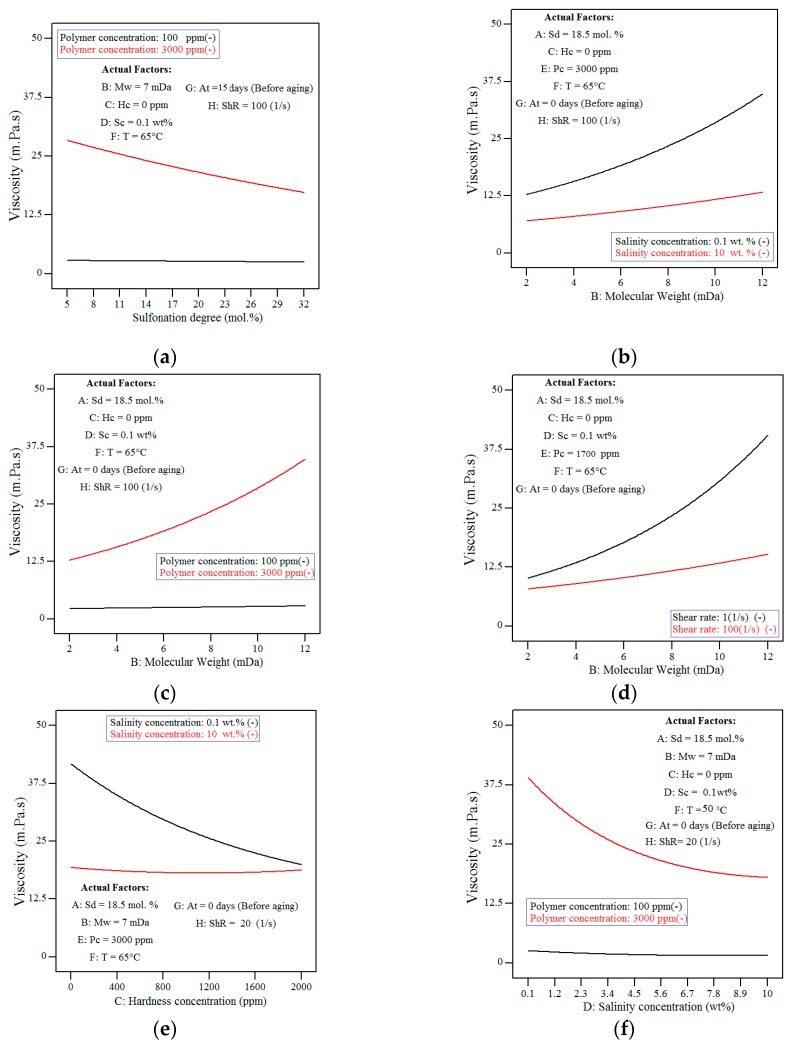

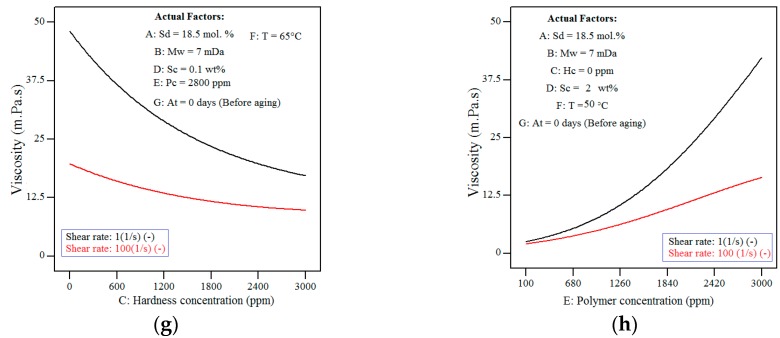
Interaction plot between model inputs, (**a**) polymer concentration and sulfonation degree, (**b**) salinity concentration and molecular weight, (**c**) polymer concentration and molecular weight, (**d**) shear rate and molecular weight, (**e**) salinity concentration and hardness concentration, (**f**) polymer concentration and salinity concentration, (**g**) shear rate and hardness concentration, (**h**) shear rate and polymer concentration.

As evidenced by Equation (12), there are interactions between the factors. The interactions between the factors can be established by using an interaction graph to display any of the two-factor interactions. In each part of Figure 9, the obvious non-parallel lines had demonstrated an interaction exists between the two factors (the details on the interaction evaluation is provided in reference no. [48]). All of the interaction terms will be highlighted and discussed in the subsequent paragraphs.

**Figure 10:**
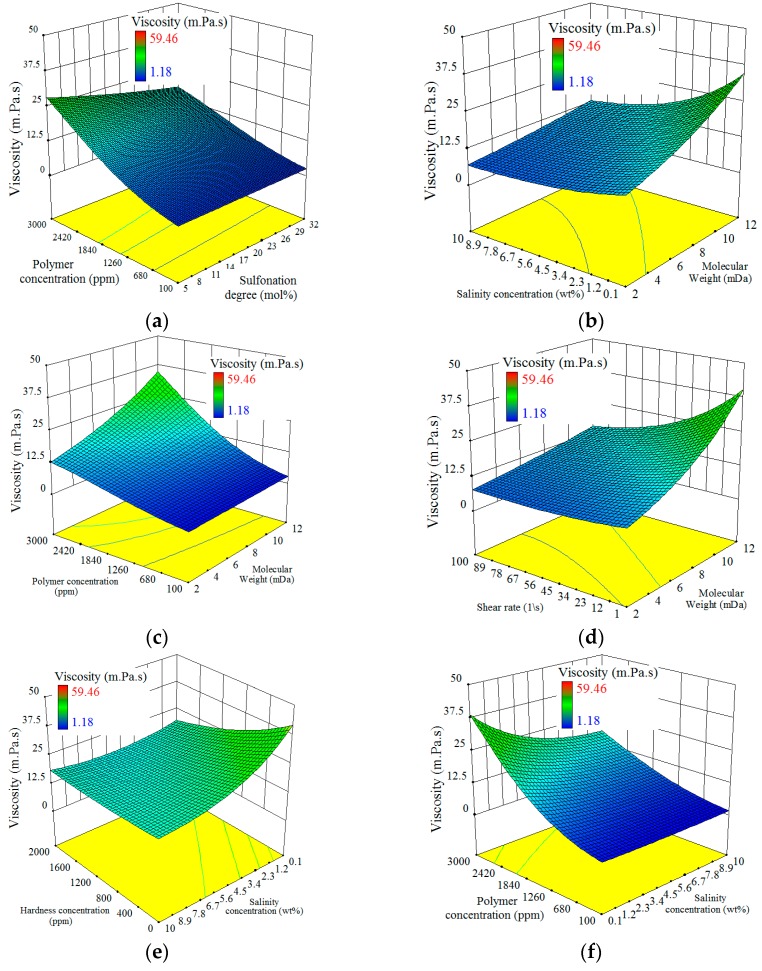

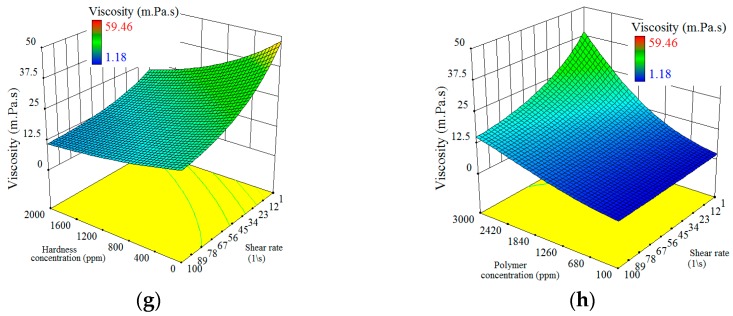
Model prediction as function of (**a**) polymer concentration and sulfonation degree, (**b**) salinity concentration and molecular weight, (**c**) polymer concentration and molecular weight, (**d**) shear rate and molecular weight, (**e**) salinity concentration and hardness concentration, (**f**) polymer concentration and salinity concentration, (**g**) shear rate and hardness concentration, (**h**) shear rate and polymer concentration.

The interaction between polymer concentration and sulfonation degree (Pc*Sd) can be observed in Figure 10a. The figure shows that increasing sulfonation degree had almost no effect on the solution viscosity when polymer concentration is low (100 ppm) in a solution with 1000 ppm salinity concentration. However, increasing sulfonation degree in a solution with 3000 ppm polymer concentration led to a lower viscosity. This interaction can be explained by the fact that at low polymer concentration (below than C*), polymer chains are not in contact with each other and therefore viscosity reduction is not significant by increasing sulfonation degree. However, chain interaction will be greatly enhanced at a high polymer concentration of 3000 ppm and lead to relatively high viscosity. Having a salinity concentration of 1000ppm in the solution results in shielding of the ${\mathrm{SO}}_{3}^{-}$ groups and therefore reduce the viscosity. The shielding of the ${\mathrm{SO}}_{3}^{-}$ groups had not only reduced the polymer hydrodynamic size but had also facilitated the CH_3_ (or hydrophobic group) association in the further reduction of the hydrodynamic size and led to the reduction of the viscosity level. Under this circumstance, increasing the sulfonation degree will tend to increase the chances of hydrophobic group associations and consequently, a reduction in the viscosity level.

Figure 10b shows the interaction between salinity concentration and molecular weight (Sc*Mw). The results had shown that the increase in salinity concentration from 0.1 wt % to 10 wt % with polymers of a low molecular weight (2 MDa) had caused a much lesser viscosity reduction than those with a higher molecular weight (12 MDa). To describe this behaviour, special consideration should be given to the hydrodynamic size of the polymer. When polymers with low molecular weight are used, the addition of a small amount of salt will result in the highest possible contraction of the polymer molecule size, where the addition of extra salts will no longer affect the size reduction. However, increasing the salinity for the polymers with high molecular weight will result in greater size reductions due to the initial large size of the molecules.

Figure 10c demonstrations the interaction between polymer concentration and molecular weights (Pc*Mw). The figure shows that the increase of 100 to 3000 ppm polymer concentration for polymers with a low molecular weight (2 MDa) had resulted in a lesser viscosity increment than those with higher molecular weights (12 MDa). Again, the hydrodynamic size of the polymer can be used to explain this phenomenon. For polymers that consist of small molecule sizes and lower molecular weights, a relatively higher polymer concentration would be required to initiate the interaction between the polymer chains, while the inverse will be applied on polymers with larger molecule sizes and higher molecular weights. When the high molecular weight polymers are used, the phase regime will change from one of “diluted” to one of “semi-diluted” and “concentrated” in much lower polymer concentration than those with low molecular weights. As discussed earlier, the changes in the regime were found to have a drastic effect on the polymer’s solution viscosity.

Figure 10d displays the interaction between the shear rate and molecular weights (ShR*Mw). The figure shows that polymers with lower molecular weights (2 MDa) had experienced a much lesser viscosity reduction than those with higher molecular weights (12 MDa), when the shear rate is increased from 1 to 100 (1/s). Basically, the main role of the shear force is to uncoil the polymer and to provide orientation and flow direction of the polymer molecules. This figure had confirmed that the increase of shear rate from 1 to 100 (1/s) had not brought any significant changes in small-sized polymers with low molecular weights as opposed to those with higher molecular weights. Hence, this had implied that low molecular weight polymers with an original small extension in the solution had not required a high shear rate for it to reach its maximum orientation level. 

Figure 10e demonstrates the interaction between salinity concentration and the hardness concentration (Sc*Hc). The figure shows that the solution viscosity had experienced a sharp drop when the salinity had changed from 0.1 to 10 wt % in the absence of divalent ions. This phenomenon, however, had not been observed at a high hardness concentration (>1800 ppm) since, at that level, all the negative charges of polymers would have already been screened by the Ca^2+^ counter ions and the hydrodynamic size of the polymers would have already reached their minimum possible value. Under this circumstance, further changes on the salinity will no longer have any effect on the hydrodynamic sizes and the viscosity of the polymer solution.

Figure 10f shows the interaction between salinity concentration and polymer concentration (Sc*Pc), where a greater effect on viscosity was observed from the increased polymer concentration in a solution with low salinity concentration than those with a higher salinity concentration. In fact, high salinity concentration leads to a low polymer hydrodynamic size which will give a lesser chance to the polymer chains to interact effectively. Sometimes due to high salinity concentration, polymer spheres are so much contracted that even increasing polymer concentration cannot result in chain interaction.

Figure 10g displays the interaction between the hardness concentration and shear rate (Hc*ShR), where there had been a greater reduction of viscosity observed from changing the shear rate from 1 to 100 (1/s) in a solution with no divalent ions as opposed to those that had contained a higher level of hardness concentration (3000 ppm). At a low hardness condition, the flexible polymer molecules would have already reached its highest possible hydrodynamic size, where further changes in the polymer deformation and orientation can be observed through the shear force application. This phenomenon can directly affect the polymer chain interaction and consequently, the viscosity level. On the contrary, a high level of hardness concentration will result in coiled polymers with a small gyration radius, where they will be less affected by shear forces and therefore resulting in solution viscosities that are more or less shear-independent. 

Figure 10h shows the interaction between polymer concentration and shear rates (Pc*ShR), where although the change of a shear rate from 1 to 100 (1/s) was found to have not caused a significant change in the viscosity level at low polymer concentration levels (100 ppm), a considerable effect on the viscosity level was, however, observed at a higher polymer concentration level. Hence, it can be concluded that the polymer concentration had played an important role in the shear rate dependency of the polymer solution. When polymer concentrations (C < C*) are low, the polymer solutions tend to behave as Newtonian fluids, that is, signifying that the viscosity is mostly shear-independent, even when the shear rate is high. In the case of higher polymer concentrations (C > C*), a decrease in viscosity was found with the rise in a shear rate like in the case for pseudo-plastic (shear thinning) fluids. This is due to the induction of polymer orientation due to the rise in shear forces that can decrease the interaction amongst polymer chains. As for the case of lower polymer concentration levels, the chains would have already been located far away from each other with almost no interaction and therefore, the effect on the polymer orientation caused by the shear force on the polymer viscosity would have been negligible.

## 5. Conclusions

This study identified and modelled the effects of significant process parameters on the bulk viscosity of sulfonated polyacrylamide polymers for the polymer-related EOR process applications. Among the process variables that had provided the greatest concern were the sulfonation degree and molecular weight (factors related to polymer characteristics); temperature, the formation of brine salinity and hardness levels (factors related to reservoir conditions); polymer concentration, shear rate as well as aging time (factors related to operational variables). Based on the above-identified significant factors, a systematic experimental plan that was based on the DOE was then designed for investigating their individual and interactive effects. As such, the specific conclusions can be summarized as below:

- An empirical model was developed for determining the viscosity of polymer solutions that can be used to select a suitable sulfonated polyacrylamide polymer in terms of sulfonation degree and molecular weight, as well as the optimal polymer solution concentration and brine salinity after taking into account the effects of reservoir temperature, formation brine salinity/hardness, aging time and shear rate account.
- This approach had provided an overview of the process factors’ impact on the polymer solution viscosity, where the results had shown the polymer concentration to be the main determinant because of its immediate effect on the polymer chain interaction.
- The DOE approach that was used to derive the empirical model was found to be a powerful tool for analysing the main effects (individual effect) and interaction effects (collectively effect) between any sets of two process variables.

## 6. Recommendations

Some of the recommendations that can be considered for future studies are as follows:

- The accuracy of the viscosity model of sulfonated polyacrylamide polymer can be improved by increasing the number of levels for each factor. Since this idea may increase the number of required experimental runs, factors with small effects such as temperature levels and aging time can be removed from the design for a temperature range of up to 80 °C.
- By utilizing the conclusions presented, future DOE-based model studies can extend the range of study by excluding the range in which a factor did not show sensitivity or the range in which the factor showed a linear or log-linear behaviour. For example, the polymers have shown to be stable up to 80 °C, which can be extended to a maximum of 90 °C by modifying the range.
- From the conclusions derived in this research, the DOE-based model studies can be extended to a wider range of study that includes the elimination of the factor range that does not exhibit sensitivity or the range in which the factor had shown a linear or log-linear behavioural trait. For example, the sulfonated polymers that were initially shown to be stable of up to 80 °C can be further extended to a maximum of 90 °C after the range modification.
- It is suggested that similar kind of DOE-based studies be conducted on other polymer types such as those of PAM (polyacrylamide), HPAM, thermo-associative and so on.

## Figures and Tables

**Table 1:** Investigation range of model input (factors).

Model Inputs (Factors) ^1^	Min ^2^	Middle ^2^	Max ^2^	Justification
Sulfonation degree (Sd), mol %	5(−1)	18.5(0)	32(1)	Range of available commercial products
Molecular weight (Mw), MDa	2(−1)	7(0)	12(1)	Range of available commercial products.
Hardness concentration (Hc), ppm	0(−1)	1500(0)	3000(1)	Hard to find a reservoir containing hardness level higher than 3000 ppm
Salinity concentration (Sc), Wt %	0.1(−1)	5.05(0)	10(1)	Majority of reservoir oil has salinity concentration in this range
Polymer Concentration (Pc), ppm	100(−1)	1550(0)	3000(1)	Apart from injectivity problems, injection of polymer with a concentration higher than 3000ppm is not economical.
Temperature (T), °C	50(−1)	65(0)	80(1)	Polymer instability for temperature higher than 80°C.
Aging Time (At), days	0(−1)	15(0)	30(1)	Experimental limitation
Shear Rate (ShR), 1/s	1(−1)	50.5(0)	100(1)	Expected shear rate in the reservoir is in the range of 1 to 20 1/s

^1^ All factors are numeric and continuous; ^2^ Values provided in the parenthesis are coded value of each actual value obtained using Equation (10).

**Table 2:** Polymer characteristics.

Polymer Product (Trade Name)	Molecular Weight (Million Daltons)	Sulfonation Degree (mol. %)	Data Source
AN105	6	5	[17,18]
AN105 VHM	12	5	Company Data
AN113 VLM	2	13	Company Data
AN113	8	13	[17,18]
AN113 VHM	12	13	Company Data
AN125 VLM	2	25	[17,18]
AN132	8	32	[17,18]
AN132 VHM	12	32	[51]

**Table 3:** Confounding evaluation using Pearson Correlation (r).

	Sd ^1^	M_w_^2^	Hc ^3^	Sc ^4^	Pc ^5^	T ^6^	At ^7^	ShR ^8^
Sd	1.000							
Mw	0.091	1.000						
Hc	0.000	0.000	1.000					
Sc	0.000	0.000	0.000	1.000				
Pc	0.000	0.000	0.000	0.000	1.000			
T	0.000	0.000	0.000	0.000	0.000	1.000		
At	0.000	0.000	0.000	0.000	0.000	0.000	1.000	
ShR	0.000	0.000	0.000	0.000	0.000	0.000	0.000	1.000

^1^ Sd: Sulfonation degree; ^2^ M_w_: Molecular weight; ^3^ Hc: Hardness concentration; ^4^ Sc: Salinity concentration; ^5^ Pc: Polymer concentration; ^6^ T: Temperature; ^7^ At: Aging Time; ^8^ ShR: Shear Rate.

**Table 4:** ANOVA for response surface reduced quadratic model (viscosity is the response and transformed using Equation (11)).

Source	Sum of Squares	df	Mean Square	F Value	*p*-Value Prob > F
Model	110.58	20	5.53	153.42	<0.0001
Sd ^a^	1.38	1	1.38	38.30	<0.0001
Mw ^b^	8.40	1	8.40	233.00	<0.0001
Hc ^c^	1.58	1	1.58	43.86	<0.0001
Sc ^d^	0.15	1	0.15	4.14	0.0446
Pc ^e^	67.98	1	67.98	1886.26	<0.0001
T ^f^	0.27	1	0.27	7.61	0.0069
At ^g^	0.12	1	0.12	3.29	0.0729
ShR ^h^	2.39	1	2.39	66.22	<0.0001
Sd*Pc	0.21	1	0.21	5.91	0.0168
Mw*Sc	0.20	1	0.20	5.67	0.0192
Mw*Pc	0.77	1	0.77	21.50	<0.0001
Mw*ShR	0.79	1	0.79	21.83	<0.0001
Hc*Sc	1.81	1	1.81	50.20	<0.0001
Hc*ShR	0.17	1	0.17	4.58	0.0348
Sc*Pc	0.12	1	0.12	3.33	0.0712
Pc*ShR	0.87	1	0.87	24.03	<0.0001
Hc^2^	0.36	1	0.36	10.08	0.0020
Sc^2^	0.89	1	0.89	24.76	<0.0001
P^2^	2.63	1	2.63	73.05	<0.0001
ShR ^2^	0.57	1	0.57	15.84	0.0001
Residual	3.57	99	0.036		
Lack of Fit	3.48	92	0.038	2.90	0.0689
Pure Error	0.091	7	0.013		
Cor Total	114.15	119			

^a^ Sulfonation degree; ^b^ Molecular weight; ^c^ Hardness concentration; ^d^ Salinity concentration; ^e^ Polymer concentration; ^f^ Temperature; ^g^ Aging Time; ^h^ Shear Rate.

**Table 5:** Values of the input parameter and associated uncertainties derived from measurements number 28 from Table S1 in Supplementary File A.

	Mean Value *	Lower *	Upper *	Variance *
Sulfonation degree (mol %)	13(−0.41)	12(−0.48 **)	14(−0.33)	0.07
Molecular weight (MDa)	8(0.2)	7(0)	9(0.4)	0.2
Hardness concentration (ppm)	1500(0)	1470(−0.02)	1530(0.02)	0.03
Salinity concentration (wt %)	5.05(0)	5.04(−0.001)	5.055(0.001)	0.001
Polymer concentration (ppm)	1550(0)	1520(−0.021)	1580(0.021)	0.034
Temperature (°C)	65(0)	64.5(−0.03)	65.5(0.03)	0.066
Aging time (days)	15(0)	14.75(−0.016)	15.25(0.016)	0.016
Shear rate (1/s)	50.5(0)	50(−0.01)	51(0.01)	0.019

* Mean and Variance values are Gaussian function parameters and Lower and Upper show the lowest and highest values for each input in Mont Carlo simulation. ** Numbers in the parenthesis shows the factor value after coding.

## Supplementary Materials

The supplementary materials are available online at https://www.mdpi.com/2073-4360/11/6/1046/s1.
