# Supplementary material for: A New Empirical Model for Viscosity of Sulfonated Polyacrylamide Polymers
Source: Polymers (Basel). 2019 Jun 14;11(6):1046. doi: 10.3390/polym11061046 (PMC6630335; doi:10.3390/polym11061046)
Supplement: Supplementary file 1 [file polymers-11-01046-s001.pdf]

SUPPLEMENTARY FILE A

EXPERIMENTAL RUNS FOR VISCOSITY MODEL

Table S1: Experimental runs for viscosity model development

	Factor 1	Factor 2	Factor 3	Factor 4	Factor 5	Factor 6	Factor 7	Factor 8	Response measured	Viscosity Model prediction	Error
Run No.	Sd[1] (mol %)	Mw[2] (mDa)	Hc[3] (ppm)	Sc[4] (wt. %)	Pc[5] (ppm)	T[6] (°C)	At[7] (Days)	ShR[8] (1/s)	Viscosity (mPa.s)	(mPa.s)	(mPa.s)
1	32(1)	8(0.2)	1500(0)	0.1(-1)	1550(0)	50(-1)	30(1)	50.5(0)	5.1	6.38	-1.28
2	13(-0.4)	8(0.2)	0(-1)	10(1)	1550(0)	65(0)	30(1)	1(-1)	11.0	11.88	-0.88
3	5(-1)	6(-0.2)	0(-1)	5.05(0)	1550(0)	80(1)	15(0)	100(1)	7.7	7.51	0.19
4	32(1)	12(1)	1500(0)	5.05(0)	1550(0)	65(0)	0(-1)	1(-1)	10.9	11.43	-0.53
5	5(-1)	6(-0.2)	1500(0)	10(1)	1550(0)	50(-1)	30(1)	50.5(0)	6.4	6.72	-0.32
6	13(-0.4)	2(-1)	3000(1)	5.05(0)	1550(0)	50(-1)	30(1)	50.5(0)	4.0	3.64	0.36
7	13(-0.4)	8(0.2)	3000(1)	10(1)	3000(1)	80(1)	15(0)	50.5(0)	20.4	22.90	-2.5
8	13(-0.4)	12(1)	1500(0)	0.1(-1)	1550(0)	50(-1)	15(0)	100(1)	8.2	8.78	-0.58
9	13(-0.4)	8(0.2)	1500(0)	5.05(0)	1550(0)	65(0)	15(0)	50.5(0)	7.9	6.39	1.51
10	32(1)	8(0.2)	3000(1)	5.05(0)	3000(1)	65(0)	30(1)	50.5(0)	11.7	11.66	0.04
11	32(1)	8(0.2)	1500(0)	10(1)	100(-1)	65(0)	15(0)	1(-1)	1.9	1.76	0.14
12	25(0.5)	2(-1)	0(-1)	10(1)	1550(0)	65(0)	15(0)	50.5(0)	3.6	4.28	-0.68
13	13(-0.4)	8(0.2)	1500(0)	5.05(0)	1550(0)	65(0)	15(0)	50.5(0)	7.1	6.39	0.71
14	13(-0.4)	2(-1)	0(-1)	5.05(0)	100(-1)	65(0)	15(0)	1(-1)	1.4	1.46	-0.06
15	5(-1)	6(-0.2)	1500(0)	10(1)	1550(0)	80(1)	0(-1)	50.5(0)	8.0	7.04	0.96
16	32(1)	12(1)	3000(1)	10(1)	1550(0)	65(0)	15(0)	50.5(0)	7.5	9.00	-1.5
17	13(-0.4)	12(1)	1500(0)	10(1)	3000(1)	65(0)	30(1)	50.5(0)	28.4	26.85	1.55
18	5(-1)	6(-0.2)	0(-1)	5.05(0)	3000(1)	65(0)	30(1)	50.5(0)	21.0	21.06	-0.06
19	5(-1)	6(-0.2)	1500(0)	0.1(-1)	1550(0)	50(-1)	0(-1)	50.5(0)	5.2	6.55	-1.35
20	13(-0.4)	12(1)	3000(1)	5.05(0)	3000(1)	65(0)	15(0)	100(1)	16.4	17.89	-1.49
21	13(-0.4)	2(-1)	3000(1)	5.05(0)	3000(1)	65(0)	15(0)	1(-1)	9.7	10.08	-0.38
22	13(-0.4)	2(-1)	0(-1)	5.05(0)	1550(0)	50(-1)	0(-1)	50.5(0)	4.0	4.64	-0.64
23	13(-0.4)	8(0.2)	0(-1)	0.1(-1)	100(-1)	50(-1)	15(0)	50.5(0)	1.8	2.58	-0.78
24	13(-0.4)	2(-1)	1500(0)	0.1(-1)	1550(0)	80(1)	15(0)	100(1)	4.5	5.42	-0.92
25	13(-0.4)	2(-1)	1500(0)	0.1(-1)	100(-1)	65(0)	0(-1)	50.5(0)	1.4	1.06	0.34
26	13(-0.4)	2(-1)	1500(0)	0.1(-1)	3000(1)	65(0)	30(1)	50.5(0)	12.8	10.50	2.3
27	25(0.5)	2(-1)	1500(0)	5.05(0)	3000(1)	50(-1)	15(0)	50.5(0)	5.8	6.13	-0.33

28	13(-0.4)	8(0.2)	1500(0)	5.05(0)	1550(0)	65(0)	15(0)	50.5(0)	6.0	6.39	-0.39
29	32(1)	8(0.2)	3000(1)	5.05(0)	1550(0)	80(1)	15(0)	100(1)	4.9	5.10	-0.2
30	13(-0.4)	12(1)	1500(0)	10(1)	100(-1)	65(0)	0(-1)	50.5(0)	1.4	1.66	-0.26
31	5(-1)	6(-0.2)	3000(1)	5.05(0)	1550(0)	50(-1)	15(0)	100(1)	5.4	5.58	-0.18
32	5(-1)	6(-0.2)	1500(0)	0.1(-1)	100(-1)	65(0)	15(0)	1(-1)	1.4	1.65	-0.25
33	32(1)	12(1)	1500(0)	5.05(0)	1550(0)	65(0)	30(1)	100(1)	6.3	5.74	0.56
34	5(-1)	6(-0.2)	1500(0)	5.05(0)	1550(0)	65(0)	0(-1)	1(-1)	7.3	7.77	-0.47
35	32(1)	8(0.2)	0(-1)	5.05(0)	3000(1)	65(0)	0(-1)	50.5(0)	10.6	14.82	-4.22
36	13(-0.4)	8(0.2)	1500(0)	5.05(0)	3000(1)	50(-1)	30(1)	1(-1)	24.9	28.65	-3.75
37	13(-0.4)	8(0.2)	1500(0)	5.05(0)	1550(0)	65(0)	15(0)	50.5(0)	7.5	6.39	1.11
38	13(-0.4)	8(0.2)	1500(0)	5.05(0)	1550(0)	65(0)	15(0)	50.5(0)	7.1	6.39	0.71
39	13(-0.4)	8(0.2)	3000(1)	0.1(-1)	100(-1)	80(1)	15(0)	50.5(0)	1.2	1.23	-0.03
40	13(-0.4)	2(-1)	0(-1)	5.05(0)	1550(0)	80(1)	30(1)	50.5(0)	5.5	5.85	-0.35
41	32(1)	12(1)	1500(0)	5.05(0)	3000(1)	80(1)	15(0)	50.5(0)	21.5	20.25	1.25
42	5(-1)	6(-0.2)	0(-1)	5.05(0)	100(-1)	65(0)	0(-1)	50.5(0)	1.5	1.61	-0.11
43	13(-0.4)	8(0.2)	0(-1)	10(1)	3000(1)	50(-1)	15(0)	50.5(0)	17.4	16.20	1.2
44	5(-1)	6(-0.2)	1500(0)	5.05(0)	1550(0)	65(0)	30(1)	100(1)	6.3	6.07	0.23
45	13(-0.4)	8(0.2)	0(-1)	0.1(-1)	1550(0)	65(0)	0(-1)	1(-1)	35.0	21.40	13.6
46	13(-0.4)	8(0.2)	3000(1)	10(1)	1550(0)	65(0)	30(1)	100(1)	6.8	9.04	-2.24
47	13(-0.4)	2(-1)	1500(0)	10(1)	1550(0)	50(-1)	15(0)	100(1)	4.9	5.13	-0.23
48	13(-0.4)	8(0.2)	1500(0)	5.05(0)	100(-1)	50(-1)	0(-1)	1(-1)	1.4	1.45	-0.05
49	5(-1)	12(1)	1500(0)	5.05(0)	1550(0)	65(0)	30(1)	1(-1)	17.8	17.11	0.69
50	13(-0.4)	12(1)	0(-1)	5.05(0)	1550(0)	50(-1)	30(1)	50.5(0)	11.8	11.34	0.46
51	5(-1)	6(-0.2)	3000(1)	10(1)	1550(0)	65(0)	15(0)	50.5(0)	6.8	8.53	-1.73
52	32(1)	8(0.2)	0(-1)	5.05(0)	100(-1)	65(0)	30(1)	50.5(0)	1.2	1.69	-0.49
53	25(0.5)	2(-1)	1500(0)	5.05(0)	100(-1)	80(1)	15(0)	50.5(0)	1.2	1.06	0.14
54	5(-1)	6(-0.2)	0(-1)	5.05(0)	1550(0)	50(-1)	15(0)	1(-1)	12.4	10.91	1.49
55	32(1)	8(0.2)	0(-1)	5.05(0)	1550(0)	80(1)	15(0)	1(-1)	11.8	11.55	0.25
56	5(-1)	6(-0.2)	1500(0)	10(1)	100(-1)	65(0)	15(0)	100(1)	1.6	1.85	-0.25
57	13(-0.4)	8(0.2)	3000(1)	0.1(-1)	3000(1)	50(-1)	15(0)	50.5(0)	17.4	15.39	2.01
58	13(-0.4)	12(1)	1500(0)	0.1(-1)	100(-1)	65(0)	30(1)	50.5(0)	2.2	2.12	0.08
59	32(1)	8(0.2)	0(-1)	5.05(0)	1550(0)	50(-1)	15(0)	100(1)	5.0	5.20	-0.2
60	13(-0.4)	2(-1)	1500(0)	0.1(-1)	1550(0)	50(-1)	15(0)	1(-1)	4.2	4.93	-0.73
61	13(-0.4)	8(0.2)	3000(1)	0.1(-1)	1550(0)	65(0)	30(1)	1(-1)	8.4	8.24	0.16
62	5(-1)	6(-0.2)	3000(1)	5.05(0)	1550(0)	80(1)	15(0)	1(-1)	7.9	7.55	0.35
63	32(1)	12(1)	0(-1)	0.1(-1)	1550(0)	65(0)	15(0)	50.5(0)	23.6	16.73	6.87
64	13(-0.4)	8(0.2)	0(-1)	0.1(-1)	1550(0)	65(0)	30(1)	100(1)	16.8	12.17	4.63
65	5(-1)	12(1)	3000(1)	0.1(-1)	1550(0)	65(0)	15(0)	50.5(0)	7.3	9.57	-2.27
66	13(-0.4)	12(1)	0(-1)	5.05(0)	3000(1)	65(0)	15(0)	1(-1)	59.5	75.52	-16.02
67	5(-1)	6(-0.2)	1500(0)	5.05(0)	100(-1)	50(-1)	15(0)	50.5(0)	1.5	1.19	0.31
68	13(-0.4)	2(-1)	0(-1)	5.05(0)	3000(1)	65(0)	15(0)	100(1)	8.1	9.23	-1.13
69	25(0.5)	2(-1)	3000(1)	0.1(-1)	1550(0)	65(0)	15(0)	50.5(0)	3.0	2.81	0.19

70	32(1)	8(0.2)	1500(0)	10(1)	1550(0)	50(-1)	0(-1)	50.5(0)	4.9	5.06	-0.16
71	5(-1)	6(-0.2)	1500(0)	0.1(-1)	3000(1)	65(0)	15(0)	100(1)	14.4	16.19	-1.79
72	5(-1)	6(-0.2)	1500(0)	5.05(0)	3000(1)	80(1)	15(0)	50.5(0)	19.3	16.41	2.89
73	25(0.5)	2(-1)	1500(0)	5.05(0)	1550(0)	65(0)	0(-1)	100(1)	3.1	3.68	-0.58
74	13(-0.4)	8(0.2)	1500(0)	5.05(0)	3000(1)	80(1)	0(-1)	1(-1)	24.4	30.01	-5.61
75	25(0.5)	2(-1)	1500(0)	5.05(0)	1550(0)	65(0)	30(1)	1(-1)	3.5	4.22	-0.72
76	13(-0.4)	2(-1)	1500(0)	10(1)	3000(1)	65(0)	0(-1)	50.5(0)	9.8	9.07	0.73
77	13(-0.4)	8(0.2)	0(-1)	0.1(-1)	3000(1)	80(1)	15(0)	50.5(0)	50.0	41.67	8.33
78	13(-0.4)	2(-1)	3000(1)	5.05(0)	100(-1)	65(0)	15(0)	100(1)	1.5	1.46	0.04
79	13(-0.4)	8(0.2)	1500(0)	5.05(0)	3000(1)	50(-1)	0(-1)	100(1)	11.4	11.16	0.24
80	32(1)	12(1)	1500(0)	5.05(0)	100(-1)	50(-1)	15(0)	50.5(0)	1.5	1.34	0.16
81	32(1)	8(0.2)	3000(1)	5.05(0)	1550(0)	50(-1)	15(0)	1(-1)	6.1	6.06	0.04
82	5(-1)	12(1)	1500(0)	5.05(0)	1550(0)	65(0)	0(-1)	100(1)	6.7	7.19	-0.49
83	13(-0.4)	8(0.2)	1500(0)	5.05(0)	1550(0)	65(0)	15(0)	50.5(0)	7.9	6.39	1.51
84	13(-0.4)	8(0.2)	1500(0)	5.05(0)	1550(0)	65(0)	15(0)	50.5(0)	6.3	6.39	-0.09
85	13(-0.4)	8(0.2)	1500(0)	5.05(0)	100(-1)	80(1)	30(1)	1(-1)	1.8	1.82	-0.02
86	13(-0.4)	8(0.2)	1500(0)	5.05(0)	100(-1)	50(-1)	30(1)	100(1)	1.6	1.42	0.18
87	13(-0.4)	8(0.2)	0(-1)	10(1)	100(-1)	80(1)	15(0)	50.5(0)	1.6	1.71	-0.11
88	13(-0.4)	2(-1)	3000(1)	5.05(0)	1550(0)	80(1)	0(-1)	50.5(0)	3.9	3.82	0.08
89	5(-1)	6(-0.2)	1500(0)	10(1)	3000(1)	65(0)	15(0)	1(-1)	40.2	26.76	13.44
90	13(-0.4)	12(1)	0(-1)	5.05(0)	1550(0)	80(1)	0(-1)	50.5(0)	11.8	11.89	-0.09
91	13(-0.4)	8(0.2)	1500(0)	5.05(0)	100(-1)	80(1)	0(-1)	100(1)	1.3	1.49	-0.19
92	13(-0.4)	8(0.2)	3000(1)	10(1)	1550(0)	65(0)	0(-1)	1(-1)	10.0	11.26	-1.26
93	5(-1)	6(-0.2)	0(-1)	0.1(-1)	1550(0)	65(0)	15(0)	50.5(0)	11.5	12.70	-1.2
94	13(-0.4)	8(0.2)	3000(1)	0.1(-1)	1550(0)	65(0)	0(-1)	100(1)	6.2	5.51	0.69
95	13(-0.4)	8(0.2)	0(-1)	10(1)	1550(0)	65(0)	0(-1)	100(1)	6.5	5.61	0.89
96	13(-0.4)	12(1)	0(-1)	5.05(0)	100(-1)	65(0)	15(0)	100(1)	1.5	1.79	-0.29
97	5(-1)	6(-0.2)	3000(1)	5.05(0)	3000(1)	65(0)	0(-1)	50.5(0)	13.9	13.84	0.06
98	13(-0.4)	2(-1)	1500(0)	10(1)	1550(0)	80(1)	15(0)	1(-1)	4.6	6.17	-1.57
99	13(-0.4)	2(-1)	1500(0)	10(1)	100(-1)	65(0)	30(1)	50.5(0)	1.8	1.45	0.35
100	13(-0.4)	12(1)	3000(1)	5.05(0)	100(-1)	65(0)	15(0)	1(-1)	1.8	1.92	-0.12
101	13(-0.4)	12(1)	1500(0)	10(1)	1550(0)	80(1)	15(0)	100(1)	8.8	7.55	1.25
102	32(1)	8(0.2)	1500(0)	10(1)	1550(0)	80(1)	30(1)	50.5(0)	5.3	6.37	-1.07
103	5(-1)	12(1)	1500(0)	5.05(0)	100(-1)	80(1)	15(0)	50.5(0)	1.6	1.76	-0.16
104	13(-0.4)	8(0.2)	3000(1)	10(1)	100(-1)	50(-1)	15(0)	50.5(0)	2.1	1.84	0.26
105	32(1)	8(0.2)	1500(0)	0.1(-1)	3000(1)	65(0)	15(0)	1(-1)	35.5	28.19	7.31
106	13(-0.4)	12(1)	1500(0)	0.1(-1)	3000(1)	65(0)	0(-1)	50.5(0)	28.6	37.39	-8.79
107	5(-1)	6(-0.2)	3000(1)	5.05(0)	100(-1)	65(0)	30(1)	50.5(0)	1.3	1.27	0.03
108	32(1)	8(0.2)	1500(0)	0.1(-1)	1550(0)	80(1)	0(-1)	50.5(0)	5.0	6.69	-1.69
109	13(-0.4)	12(1)	3000(1)	5.05(0)	1550(0)	50(-1)	0(-1)	50.5(0)	9.2	7.43	1.77
110	13(-0.4)	12(1)	1500(0)	10(1)	1550(0)	50(-1)	15(0)	1(-1)	13.0	14.30	-1.3
111	32(1)	8(0.2)	3000(1)	5.05(0)	100(-1)	65(0)	0(-1)	50.5(0)	1.3	1.10	0.2

112	5(-1)	12(1)	1500(0)	5.05(0)	3000(1)	50(-1)	15(0)	50.5(0)	18.4	28.94	-10.54
113	5(-1)	12(1)	0(-1)	10(1)	1550(0)	65(0)	15(0)	50.5(0)	12.2	9.98	2.22
114	13(-0.4)	8(0.2)	1500(0)	5.05(0)	1550(0)	65(0)	15(0)	50.5(0)	6.0	6.39	-0.39
115	32(1)	8(0.2)	1500(0)	10(1)	3000(1)	65(0)	15(0)	100(1)	11.3	9.08	2.22
116	13(-0.4)	8(0.2)	1500(0)	5.05(0)	3000(1)	80(1)	30(1)	100(1)	14.8	14.02	0.78
117	13(-0.4)	12(1)	1500(0)	0.1(-1)	1550(0)	80(1)	15(0)	1(-1)	27.3	21.86	5.44
118	13(-0.4)	12(1)	3000(1)	5.05(0)	1550(0)	80(1)	30(1)	50.5(0)	9.3	9.34	-0.04
119	5(-1)	6(-0.2)	1500(0)	0.1(-1)	1550(0)	80(1)	30(1)	50.5(0)	5.3	8.24	-2.94
120	32(1)	8(0.2)	1500(0)	0.1(-1)	100(-1)	65(0)	15(0)	100(1)	1.4	1.58	-0.18

Note: Values provided in the parenthesis are coded value of each actual value using Equation **Error! Reference source not found.**).

[1]Sulfonation degree; [2]Molecular weight; [3]Hardness concentration;[4]Salinty concentration;[5]Polymer concentration; [6]Temperature;[7]Aging Time; [8]Shear Rate.

SUPPLEMENTARY FILE B

SIMPLIFIED VISCOSITY MODELS

Table S1: Simplified viscosity models driven from Equation **Error! Reference source not found.**).

Removed term*	Resulting Model**	Error Evaluation
Sc*Pc	$Ln\left(\frac{\mu}{1000-\mu}\right) = -5.21 - 0.16A + 0.40B - 0.17$ $-0.052D + 1.20E + 0.070F + 0.046G - 0.21H$ $-0.094AE - 0.095BD + 0.20BE - 0.19\,BH + 0.27CD$ $+0.088CH - 0.19EH + 0.11C^2 + 0.17D^2 - 0.30E^2$ $+0.14H^2$	R-Squared 0.9677<br>Adj R-Squared 0.9616<br>Pred R-Squared 0.9522<br>Adeq Precision 55.683
Hc*ShR	$Ln\left(\frac{\mu}{1000-\mu}\right) = -5.21 - 0.16A + 0.40B - 0.17C$ $-0.052D + 1.19E + 0.069F + 0.046G - 0.21H$ $-0.098AE - 0.095BD + 0.23BE - 0.19\,BH + 0.27CD$ $-0.19EH + 0.11C^2 + 0.17D^2 - 0.30E^2 + 0.14H^2$	R-Squared 0.9662<br>Adj R-Squared 0.9602<br>Pred R-Squared 0.9511<br>Adeq Precision 54.953
Mw*Sc	$Ln\left(\frac{\mu}{1000-\mu}\right) = -5.21 - 0.16A + 0.40B - 0.17C$ $-0.060D + 1.19E + 0.069F + 0.046G - 0.21H$ $-0.098AE + 0.23BE - 0.19\,BH + 0.27CD - 0.20EH$ $+0.11C^2 + 0.17D^2 - 0.30E^2 + 0.14H^2$	R-Squared 0.9645<br>Adj R-Squared 0.9585<br>Pred R-Squared 0.9498<br>Adeq Precision 55.408
Sd*Pc	$Ln\left(\frac{\mu}{1000-\mu}\right) = -5.21 - 0.16A + 0.40B - 0.17C$ $-0.060D + 1.22E + 0.068F + 0.046G - 0.21H$ $+0.22BE - 0.19\,BH + 0.27CD - 0.20EH + 0.11C^2$ $+0.17D^2 - 0.30E^2 + 0.14H^2$	R-Squared 0.9625<br>Adj R-Squared 0.9566<br>Pred R-Squared 0.9485<br>Adeq Precision 56.928
At	$Ln\left(\frac{\mu}{1000-\mu}\right) = -5.21 - 0.16A + 0.40B - 0.17C$ $-0.060D + 1.22E + 0.068F - 0.21H + 0.22BE$ $-0.19\,BH + 0.27CD - 0.20EH + 0.11C^2 + 0.17D^2$ $-0.30E^2 + 0.14H^2$	R-Squared 0.9614<br>Adj R-Squared 0.9559<br>Pred R-Squared 0.9480<br>Adeq Precision 57.709
T	$Ln\left(\frac{\mu}{1000-\mu}\right) = -5.21 - 0.16A + 0.40B - 0.17C$ $-0.060D + 1.22E - 0.21H + 0.22BE - 0.19\,BH$ $+0.27CD - 0.20EH + 0.11C^2 + 0.17D^2 - 0.30E^2$ $+0.14H^2$	R-Squared 0.9592<br>Adj R-Squared 0.9537<br>Pred R-Squared 0.9460<br>Adeq Precision 59.115

Hc^2	$$Ln\left(\frac{\mu}{1000-\mu}\right) = -5.21 - 0.16A + 0.40B - 0.17C$$ $$-0.060D + 1.22E - 0.21H + 0.22BE - 0.19\,BH$$ $$+0.28CD - 0.20EH + 0.17D^2 - 0.31E^2 + 0.13H^2$$	R-Squared 0.9560<br>Adj R-Squared 0.9506<br>Pred R-Squared 0.9431<br>Adeq Precision 58.062

ShR^2	$$Ln\left(\frac{\mu}{1000-\mu}\right) = -5.07 - 0.16A + 0.40B - 0.17C$$ $$-0.060D + 1.22E - 0.21H + 0.22BE - 0.19\,BH$$ $$+0.28CD - 0.20EH + 0.16D^2 - 0.32E^2$$	R-Squared 0.9516<br>Adj R-Squared 0.9462<br>Pred R-Squared 0.9387<br>Adeq Precision 55.998
Sc^2	$$Ln\left(\frac{\mu}{1000-\mu}\right) = -5 - 0.16A + 0.40B - 0.17C$$ $$-0.060D + 1.22E - 0.21H + 0.22BE - 0.19\,BH$$ $$+0.28CD - 0.20EH - 0.33E^2$$	R-Squared 0.9453<br>Adj R-Squared 0.9398<br>Pred-Squared 0.9317<br>Adeq Precision 55.112
Mw*ShR	$$Ln\left(\frac{\mu}{1000-\mu}\right) = -5 - 0.16A + 0.40B - 0.17C$$ $$-0.060D + 1.22E - 0.22H + 0.22BE + 0.28CD$$ $$-0.20EH - 0.33E^2$$	R-Squared 0.9383<br>Adj R-Squared 0.9326<br>Pred R-Squared 0.9236<br>Adeq Precision 52.366
Pc*ShR	$$Ln\left(\frac{\mu}{1000-\mu}\right) = -5 - 0.16A + 0.40B - 0.17C$$ $$-0.060D + 1.22E - 0.22H + 0.22BE + 0.28CD$$ $$-0.33E^2$$	R-Squared 0.9301<br>Adj R-Squared 0.9244<br>Pred R-Squared 0.9149<br>Adeq Precision 51.862
Mw*Pc	$$Ln\left(\frac{\mu}{1000-\mu}\right) = -5 - 0.16A + 0.40B - 0.17C$$ $$-0.060D + 1.24E - 0.22H + 0.28CD - 0.33E^2$$	R-Squared 0.9202<br>Adj R-Squared 0.9145<br>Pred R-Squared 0.9048<br>Adeq Precision 51.873
Hc*Sc	$$Ln\left(\frac{\mu}{1000-\mu}\right) = -4.99 - 0.16A + 0.41B - 0.17C$$ $$-0.060D + 1.24E - 0.22H - 0.33E^2$$	R-Squared 0.9043<br>Adj R-Squared 0.8983<br>Pred R-Squared 0.8883<br>Adeq Precision 50.669
Sc	$$Ln\left(\frac{\mu}{1000-\mu}\right) = -4.99 - 0.16A + 0.41B - 0.17C$$ $$+1.24E - 0.22H - 0.33E^2$$	R-Squared 0.9025<br>Adj R-Squared 0.8974<br>Pred R-Squared 0.8889<br>Adeq Precision 53.910
Sd	$$Ln\left(\frac{\mu}{1000-\mu}\right) = -4.96 + 0.39B - 0.17C + 1.24E$$	R-Squared 0.8906

	$-0.22H - 0.33E^2$	Adj R-Squared 0.8858<br>Pred R-Squared 0.8776<br>Adeq Precision 54.801
Hc	$Ln\left(\frac{\mu}{1000-\mu}\right) = -4.96 + 0.39B + 1.24E - 0.22H$<br>$-0.33E^2$	R-Squared 0.8763<br>Adj R-Squared 0.8720<br>Pred R-Squared 0.8649<br>Adeq Precision 51.919
ShR	$Ln\left(\frac{\mu}{1000-\mu}\right) = -4.96 + 0.39B + 1.24E - 0.33E^2$	R-Squared 0.8516<br>Adj R-Squared 0.8478<br>Pred R-Squared 0.8415<br>Adeq Precision 46.807
Pc^2	$Ln\left(\frac{\mu}{1000-\mu}\right) = -5.11 + 0.40B + 1.24E$	R-Squared 0.8235<br>Adj R-Squared 0.8205<br>Pred R-Squared 0.8146<br>Adeq Precision 49.854
Mw	$Ln\left(\frac{\mu}{1000-\mu}\right) = -5.07 + 1.24E$	R-Squared 0.7519<br>Adj R-Squared 0.7498<br>Pred R-Squared 0.7441<br>Adeq Precision 39.144

* Sd: Sulfonation degree; Mw: Molecular weight; Hc: Hardness concentration; Sc: Salinity concentration; Pc: Polymer concentration; T: Temperature; At: Aging time; ShR: Shear rate.

** A: Sulfonation degree; B: Molecular weight; C: Hardness concentration; D: Salinity concentration; E: Polymer concentration; F: Temperature; G: Aging time; H: Shear rate.

SUPPLEMENTARY FILE C

THE DIAGNOSTIC PLOTS AS USEFUL TOOLS FOR ENSURING THE FULFILLMENT OF THE ANOVA ASSUMPTIONS

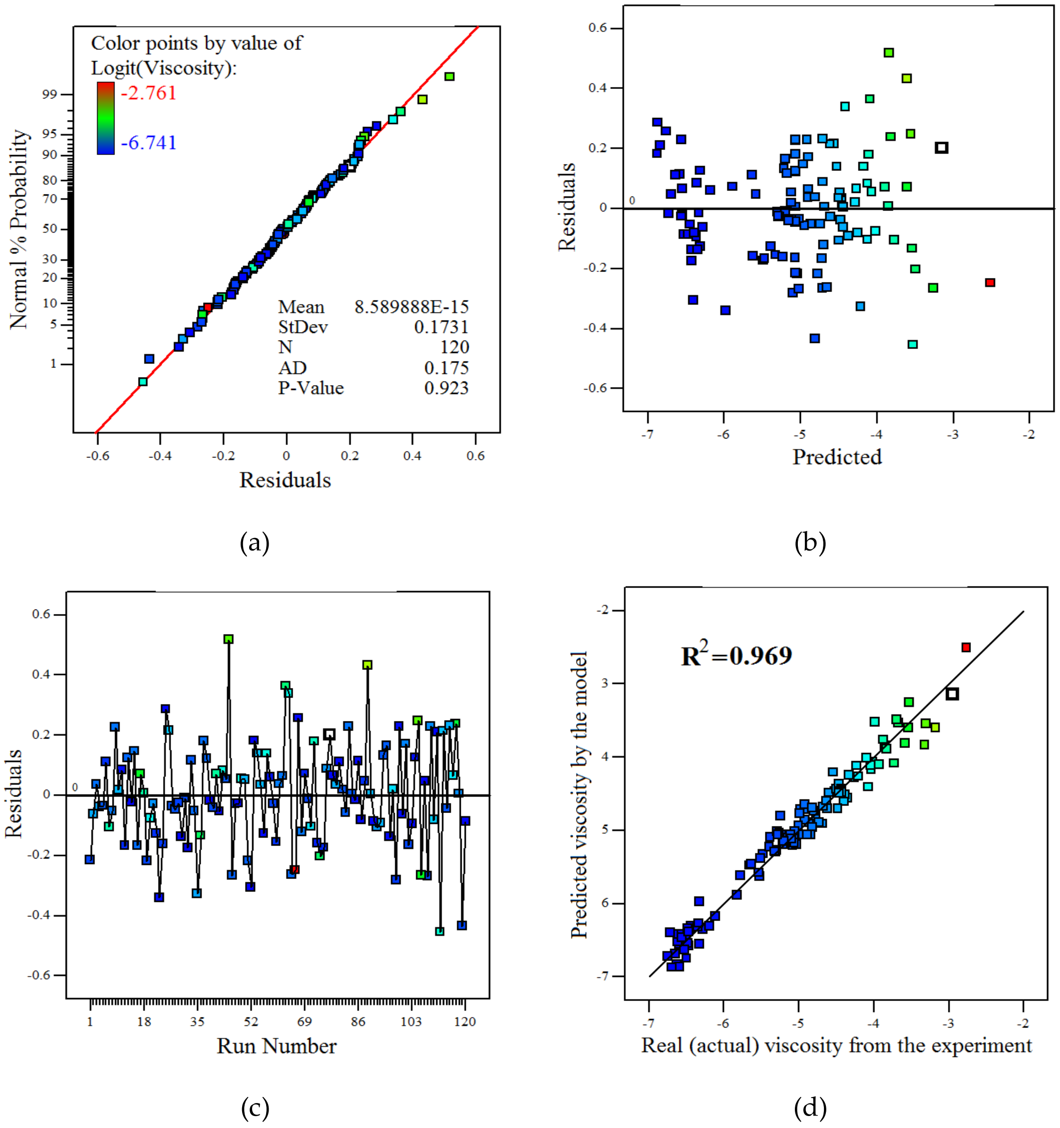

Figure S1. Diagnostics plots. Normal probability plot of the residuals (a), residuals versus predicted values (b), residuals versus run order (c), real (actual) viscosity values versus predicted viscosity based on the transformed values.

Figure C.1 of Supplementary File C shows four of these diagnostic plots which confirms that all of the assumptions required in the validation of the regression model seemed to have been fulfilled:

Part (a): Normality of residuals was assessed via a normal probability plot. The residuals' normal plot demonstrates that the plotted points fall almost on a straight line, such that the residuals formed are at least approximately normal or normal.

Part (b): Constant error is checked via residuals versus predicted values. Based on the plot of residuals versus the fitted values, residuals were seen to possess almost the same amount of variation applicable to all levels of the fitted value, i.e., signifying that residuals are homoscedastic in terms of the fitted values.

Part (c): The residuals versus run order is plotted to detect the outliers or influential values. The residuals were observed to have a uniform distribution but had been random at about Residuals= 0 hence, implying that the residuals had been homoscedastic with regards to time. As there had been no patterns to allow the prediction of a residual from those that had preceded it, these errors were then regarded as independent factors.

Part (d): Observed (actual) response values versus predicted response values distinguish a value, or a group of values, which the model could not predict easily. The 45-degree line can be employed to split data points evenly. This figure shows that the developed model is ample, since the residuals are small in each response prediction, with the residuals seemingly being close to the diagonal line.
